# The Effectiveness of Physical Literacy Interventions: A Systematic Review with Meta-Analysis

**DOI:** 10.1007/s40279-022-01738-4

**Published:** 2022-08-22

**Authors:** Johannes Carl, Jaime Barratt, Philipp Wanner, Clemens Töpfer, John Cairney, Klaus Pfeifer

**Affiliations:** 1 Department of Sport Science and Sport, Friedrich-Alexander University Erlangen-Nürnberg, Gebbertstraße 123b, 91058 Erlangen, Germany; 2 School of Human Movement and Nutrition Sciences, The University of Queensland, Brisbane, QLD 4072 Australia; 3 Institute of Sports and Sports Sciences, Ruprecht Karl University of Heidelberg, Im Neuenheimer Feld 700, 69120 Heidelberg, Germany; 4 Institute of Sports Science, Friedrich Schiller University Jena, Seidelstraße 20, 07749 Jena, Germany

## Abstract

**Background:**

The holistic concept of physical literacy assumes that individuals require adequate cognitive (knowledge and understanding), affective (motivation and confidence), and physical (physical competence) qualities to engage in lifelong physical activity behavior. In recent years, the research field has undergone rapid development and has also yielded an increasing number of interventions that aim to translate the theoretical-philosophical ideas into practical endeavors.

**Objective:**

The goal of the present pre-registered systematic review was to (a) provide a general overview of evaluation studies on physical literacy interventions and (b) to quantitatively examine the effectiveness of physical literacy interventions.

**Methods:**

Drawing on the 2020 Preferred Reporting Items for Systematic Reviews and Meta-Analyses (PRISMA) guidelines, we searched a total of 18 databases for physical literacy interventions. Inclusion criteria were English language, publication by November 2021, and interventions using physical literacy as a theoretical underpinning or evaluation outcome. Articles that met these criteria were analyzed with respect to their basic delivery characteristics, study quality, evaluation approach, and main findings. We additionally ran meta-analyses with all non-randomized and randomized controlled trials to examine and compare the effect of these interventions on five outcome categories: (i) physical competence, (ii) motivation and confidence, (iii) knowledge and understanding, (iv) physical activity behavior, and (v) total physical literacy. Standardized mean differences (SMDs) with 95% confidence intervals (CIs) were calculated to assess the effects on the different categories.

**Results:**

The screening process with two independent raters yielded 48 eligible interventions reported in 51 eligible articles. Quantitative evaluations most frequently addressed physical competence (72.2%), followed by motivation and confidence (47.2%), physical activity behavior (41.7%), and knowledge and understanding (33.3%). The controlled intervention studies (*n* = 24) exerted significant effects on all five physical literacy categories. Despite meaningful heterogeneity across the subgroups, the strongest effects were found for physical competence (SMD 0.90; 95% CI 0.55–1.25), followed by physical literacy aggregate scores (SMD 0.61; 95% CI 0.20–1.01), knowledge and understanding (SMD 0.54; 95% CI 0.30–0.79), physical activity behavior (SMD 0.39; 95% CI 0.23–0.55), and motivation and confidence (SMD 0.30; 95% CI 0.17–0.44).

**Conclusions:**

The present study empirically demonstrated the effectiveness of physical literacy interventions on several outcomes relevant for promoting physical activity and health. To better inform current practices, future studies are advised to identify those program characteristics that significantly influence the effectiveness of physical literacy interventions.

**Clinical Trial Registration:**

PROSPERO CRD42020188926.

**Supplementary Information:**

The online version contains supplementary material available at 10.1007/s40279-022-01738-4.

## Key Points

**Table:** 

Evaluation studies unequally considered the different physical literacy domains (in favor of the physical domain); affective, behavioral, and (in particular) cognitive indicators should be integrated more systematically.
The effect sizes did not significantly differ between randomized and non-randomized controlled trials.
The interventions exerted significant effects on all main outcomes of physical literacy; however, the strongest effects were found for physical outcomes, followed by (in descending order) cognitive, behavioral, and affective outcomes.

## Introduction

### Physical Literacy

Underpinned by strong and robust scientific evidence underlining the high prevalence of physical inactivity worldwide, there is currently consensus that individuals of all ages should be familiarized with physically active lifestyles [1]. Depending on the focus of research, scientists argue from a physical [2, 3], social [4], psychological [5], or general health [6] perspective why it is essential to promote people’s levels of physical activity (PA). In this context, it has been increasingly suggested to draw on the conceptualizations of physical literacy (PL) for describing the lifelong familiarization process of individuals [7]. As such, high PL levels are assumed to be positively associated with aspects of health [8–10]. Grounding on the pioneering descriptions of Margaret Whitehead [11–13], the International Physical Literacy Association (IPLA) defines PL as the “motivation, confidence, physical competence, knowledge, and understanding to value and take responsibility for engagement in physical activities for life” [14]. Even though, or perhaps because, PL has been acknowledged as a valuable concept across the world [15, 16], there are a variety of PL definitions with different emphases and weights placed on certain PL aspects. For instance, the Australian framework is very comprehensive and lists a total of 30 elements attributable to physical, psychological, social, and cognitive domains of PL [17]. In particular, the framework transcends previous conceptualizations of PL with a social aspect [18, 19]. While Canada has largely adopted the IPLA definition of PL [20], the definition in New Zealand incorporated a spiritual element [21]. On the one hand, such country-specific conceptualizations may promote conceptual diversity, stimulate tolerance, and better account for the cultural sensitivity of bodily practices [22, 23], such as cultivated, for instance, by indigenous people [24]. On the other hand, such inconsistencies mean that both researchers and practitioners often talk about slightly different concepts despite using the same terminology [25]. Therefore, studies should be explicit about their perspective on PL. Specifically, the present endeavor draws on the IPLA [14] definition of PL with its affective (motivation and confidence), physical (physical competence), and cognitive (knowledge and understanding) requirements for PA behavior (engagement in physical activities for life) [26, 27]. This definition represents the smallest number of globally shared components worldwide (variations consistently go beyond these components) while maintaining the core meaning of the concept [28]. In addition, the IPLA definition is in line with a previous analysis [29], which enables direct comparisons at a higher analytical level.

Despite the lack of a universally accepted gold standard definition and the fact that the concept has complex philosophical roots (e.g., phenomenology, existentialism) [30], PL is highly attractive for practical endeavors [16, 31]. The main reason lies in the fact that PL cultivates a holistic and integrative understanding of human movement. Framed by philosophic assumptions of monism and descriptions of embodiment [30, 32], discussions call for simultaneously addressing people’s physical, cognitive, and motivational determinants for PA [33, 34]. These determinants, in turn, have the potential to interact dynamically and reinforce reciprocally, ideally resulting in a virtuous cycle [8, 9, 35] as part of a “beatific narrative” [36]. In addition, the popularity of the PL approach can be explained by the inclusive character of the concept [32]. In line with the descriptions of a “lifelong journey” [16, 37], PL can basically be applied to all ages, spanning children [35, 38] and older adults [39]. The inclusive character also refers to individuals with developmental disorders and disabilities [40–42], turning PL into a concept for everyone.

### PL Interventions: An Effectiveness Perspective

In general, it is assumed that PL can be nourished through a range of experiences [43, 44]. In this regard, practitioners such as educators, teachers, exercise and fitness instructors, therapists, and health consultants are often given the responsibility to create situations (e.g., through specific methods) that systematically build and enhance PL. Accordingly, PL has been adopted politically in strategic concepts for PA promotion and physical education. For instance, the UNESCO [45] has highlighted PL within the Quality Physical Education Guidelines for Policy Makers. In addition, the World Health Organization calls within its Global Action Plan on PA 2018–30 for targeting PL [1]. Importantly, it has been suggested to more strongly highlight the value of PL for achieving the UN Sustainable Development Goals [46]. In light of these assumptions, well-designed interventions with the goal to enhance PL move into the focus of interest [29, 47–50]. In this context, research with a focus on interventional issues can help bridge the gap between theory and practice, improving the translation of the complex theoretical foundations, including the philosophic tenets, into effective practices is highly welcome [31, 51].

To provide solid recommendations regarding the arrangement of interventions, a research method should be chosen that builds on the experience with different target groups, intervention modalities, and implementation conditions. Accordingly, it is valuable to transcend the horizon of a single study through the accumulation of findings across different, often heterogeneous, efforts. In this context, reviews have the potential to capture studies relevant for a field, subsequently drawing profound conclusions [52, 53]. Indeed, specific to PL, there is a review of children and adolescents but it only includes a small section on interventions [38]. Saxena and Shikako-Thomas [42] analyzed PL interventions for children with disabilities by applying a realist review approach, yet the authors concluded that explaining intervention mechanisms and deriving solid recommendations is barely possible. In contrast, McKay et al. [48] dealt with intervention strategies in adults but only included two non-randomized controlled trials. Detaching from a narrow population-specific focus, a recent systematic review (which was previously performed by our team) attempted to embrace the plentitude of PL interventions and finally encompassed a total of 46 studies [29]. However, this study concentrated on the design and content of these interventions, which characterizes this review as more of a descriptive endeavor. What has been neglected thus far is a broad evaluation and effectiveness perspective on PL interventions.

Against the background of this shortcoming, important questions regarding PL interventions cannot yet be answered. For instance, this refers to the question of whether interventions are successful in affecting PL elements or outcomes relevant for PA and health. Given the positive effects achievable by interventions on constructs such as physical competence [54], knowledge [55], and motivation [56], it can be hypothesized that interventions manage to significantly influence the domains of PL (physical competence, knowledge and understanding, motivation and confidence). Moreover, it can be postulated that PL interventions should succeed in promoting physically active lifestyles (the domain “daily behavior”) when systematically addressing the determinants as mentioned within the IPLA [14] definition. In this regard, PL could be in line with other theoretical frameworks that have been shown to positively enhance levels of PA [57]. Importantly, an effectiveness perspective on PL could provide empirical arguments for enriching the interdisciplinary idea inherent to research on sport, exercise, and PA [58].

### Aims and Research Questions

The goal of this review was two-fold. First, we intended to provide a broad overview of evaluation studies on PL interventions. Complementary to the first publication of this project adopting a specific perspective on aspects of intervention design and content [29], the focus of the present analysis was placed on study quality, the evaluation approach, and the main findings. Second, we aimed to quantitatively analyze the effectiveness of PL interventions by means of meta-analytical procedures. Accounting for the multi-dimensional nature of the PL concept, we thereby intended to differentially examine the effect of these interventions in accordance with the different, yet intertwined domains of PL [59]. More specifically, the structure of the results was guided by the differentiation into the following outcome categories: physical competence, knowledge and understanding, motivation and confidence, PA, and total PL score.

## Methods

### Rationale for the Methodological Approach

Among the different types of reviews [52], a systematic review applies “systematic and explicit methods to identify, select, and critically appraise relevant research, and to collect and analyze data from the studies that are included in the review” [60]. Ideally, systematic reviews are combined with meta-analytical techniques to yield a quantitative summary of results [53]. Taken together, this methodological interplay can serve to “identify problems in primary research that should be rectified in future studies” [53]. In this regard, the selected approach has the potential to illuminate strengths and weaknesses of current PL interventions, thus contributing to improve the knowledge translation as mentioned above. This review project has been pre-registered (registration number: CRD42020188926) in the International Prospective Register of Systematic Reviews (PROSPERO) and specified within a publicly available protocol (https://www.crd.york.ac.uk/prospero/display_record.php?RecordID=188926). Because of the volume of data extracted, the present article concentrated on the quantitative effectiveness of PL interventions. The descriptive dimensions of the first part of this review project [29] informed the analyses of the present study. The review adhered to the 2020 guidelines of the Preferred Reporting Items for Systematic Reviews and Meta-Analyses (PRISMA) [53] (for the PRISMA checklist, see Table 1 of the Electronic Supplementary Material [ESM]).

### Search Process

We performed electronic searches in the following 18 databases: APA PsycARTICLES APA PsycINFO, Psychology and Behavioral Sciences Collection, SPORTDiscus, Teacher Reference Center (all via EBSCOhost), ASSIA, ERIC, IBSS, Social Services Abstracts, Sociological Abstracts, Sports Medicine and Education Index (all via ProQuest), CINAHL, Cochrane Central Register of Controlled Trials (CENTRAL), EMBASE, PubMed/MEDLINE, ScienceDirect, Scopus, and Web of Science. Two systematic reviews exerting a large impact on PL literature [16, 61] guided this decision complemented by further databases crucial for the field. The final combination of search terms adopted Boolean rules (under inclusion of truncations) and listed the theoretical approach (i.e., PL), on the one hand, and words for active ingredients (e.g., intervention, program, training), on the other hand. Table 2 of the ESM visualizes the successive development process, including the final search term combination.

The search hits (last update undertaken on 10 November, 2021) were exported into the reference management software EndNote, version X9.3.3, Clarivate Analytics. After the automatic and manual removal of duplicates, the literature entries were subject to a multi-step screening process (title, abstract, and full-text screening) in which two independent reviewers (JC, JB) rated the eligibility of each article. At each stage, the research team discussed the eligibility criteria and anticipated potential conflicts in the evaluation process. Subsequent to evaluating the articles of authors with the last names beginning with A–D (corresponding to approximately 15% of the articles), the two assessors met for specifying and refining the criteria mentioned above. For example, the specification referred to the handling of validation articles (title screening), the required characteristics of multicomponent interventions (abstract screening), and to the relevance of PL within the intervention studies (full-text screening).

### Eligibility Criteria

This systematic review only comprised intervention studies that (I) use PL as a major theoretical underpinning (operationalized as the presentation of a definition or a conceptual discussion plus a rationale for its use) *or/and* as an outcome (for a similar strategy, see [62]). Furthermore, the present review exclusively included intervention studies that (II) report any evaluation aspect, (III) were published in a scientific journal or book section, and (IV) were written in the English language. For articles to be included into the meta-analytical calculations, interventions additionally had to (V) provide quantitative data eligible for quantitative synthesis and (VI) contain at least one control group. We did not consider literature syntheses (i.e., systematic reviews or meta-analyses), conference contributions, theses, and articles in other languages than English. Moreover, we excluded naturalistic interventions without specification of content. There were no restrictions applied regarding publication date, peer-review requirements, study design, study quality, and the populations involved. Kappa values were computed for each screening stage under consideration of corresponding interpretation guidelines [63]; these statistical values were also utilized for the internal discussion (e.g., on screening quality) after each screening stage.

### Data Extraction

Two assessors (JC, JB) collaborated to extract the data and shared the process equally. While one person extracted data, the other checked the extraction process. The primary assessor (JC), in turn, had to check and approve the corrections. We designed a structured data extraction template with the following basic information on the interventions: publication date, country, setting, and population (e.g., age, sex, target group), intervention delivery (e.g., intervention focus, length, deliverer), theoretical underpinnings (e.g., PL definition, PL domains addressed), intervention content (e.g., components, link to theory) as well as study quality and intervention evaluation (see next section).

We employed items 7–11 of the Theory Coding Scheme to specify whether/how PL theory was linked to intervention content [64]. Based on this information, we subsequently coded whether intervention content was just loosely dictated by PL (“theory inspired” [65]), whether intervention components were directly derived from PL theory (“theory based” [65]), or whether the theoretical underpinnings were insufficient (meaning that the article was excluded if PL did not serve as an outcome). For this task, two raters (JC, KB) coded the studies independently and achieved an agreement rate of 83.8% (disagreements were resolved by discussion). Informed by Cochrane Collaboration’s tool for assessing the *risk of bias* [66, 67], we filtered the following information from the primary articles by coding a “yes” (criterion given) or “no” (criterion not given): (a) integration of a control group; (b) randomization; (c) multiple measurements (at both pre and post); (d) blinding; (e) description of the completeness of outcome data (e.g., attrition, exclusion, or dropout); (f) adequate handling of incomplete data (e.g., intention-to-treat paradigm, imputation techniques); (g) no suggestion of selective outcome reporting; and (h) reliable measurement of outcomes. In addition, we scanned the primary articles for quantitative information relevant for determining the *effectiveness* of the interventions included. This extraction contained the name of a construct, the measurement instrument, the timing of an assessment (e.g., before/after the intervention, follow-up), quantitative mean values and standard deviations for the intervention and the control group, the importance of values within a study (primary vs secondary outcome), as well as potential effect sizes and change scores (i.e., standardized or unstandardized). After the extraction of raw data, we assigned all outcomes to five main categories, reflecting the four PL domains (physical competence, knowledge and understanding, motivation and confidence, PA behavior) [59] and an overall PL score (defined as aggregating values or covering phenomena across at least three different PL domains). Because of considerable conceptual heterogeneity, we additionally formed subgroups after familiarization with the extracted PL indicators (for further explanations, see Table 3 of the ESM): three subcategories for the physical competence domain (fundamental movement skills; cardiorespiratory fitness; agility and lower body strength), two subcategories for the knowledge and understanding domain (objective knowledge; subjective understanding and attitude), as well as three subcategories for the motivation and confidence domain (motivation; confidence and self-efficacy; enjoyment and positive affect). The basic effectiveness was analyzed *within* the subcategories, *between* the subcategories, and *between* the five main categories. Data management was undertaken in Excel and SPSS, version 25 (IBM Corporation, Armonk, New York, USA).

### Data Synthesis (Meta-Analysis)

For estimating treatment effects, we entered post-test results (continuous data) as mean values with standard deviations for the intervention and the control group, respectively. In all outcomes, positive values indicate improvements. Negatively pooled constructs (e.g., running time representing agility) were inverted by switching the values of both groups or by multiplying the mean values by minus one. We transformed post-test values of both intervention and control groups into standardized mean differences (SMDs) and respective 95% confidence intervals (CIs). In the case of significant (*p* < 0.05) or marginally significant (*p* < 0.10) baseline differences, as indicated in the results section of a study, we compared standardized change scores instead or adjusted for pre-intervention values to minimize bias through initial differences. In the latter case, we computed the baseline-controlled effect size via the Psychometrica tool (operation 3 for pre-post-control designs) and the suggested subtraction-based calculation procedure by Klauer [68]. Afterwards, we manually adjusted the control group mean in the software in correspondence with the calculated baseline-controlled effect size (for details, see Table 4 of the ESM). If studies reported results of more than one construct per category (e.g., two different tests for fundamental movement skills), we attempted to identify the test with the most appropriate representation of this category ( “marker construct”) or generated an averaged effect size across different outcomes (for details, see Table 4 of the ESM). In line with the hierarchical structure of the outcome coding (as reported in the previous section), we performed a main analysis as well as separate subgroup analyses of the data. In our main analysis, we aimed at testing the effect of PL interventions on the level of the five superior categories (four PL domains, overall PL score). Accounting for the conceptual breadth of the main categories, the subgroup analyses aimed at specifically analyzing and comparing the effect of the interventions on the different subcategories of the domains (e.g., the three subcategories of the motivation and confidence domain). To acquire robust findings from subgroup analyses, the Cochrane handbook suggests that *k* ≥ 5 samples should be available for each category [69]. Following an exploratory approach, we decided to also conduct analyses with a smaller number of samples (*k* ≥ 3) because of its potential to inspire future investigations (for a similar strategy, see [70]). In this context, however, it should be noted that such a procedure needs to be met with caution, deriving only slight tendencies. The comparison between categories was based on both inferential statistics (via *χ*^2^ statistics and a significance level of *p* < 0.05) and heterogeneity information [69]. Heterogeneity was determined quantitatively via the *I*^2^ coefficient, with values ≥ 30% indicating meaningful subgroup differences [69]. Given the conceptual and statistical heterogeneity between studies, data of all analyses were pooled using random-effects models. We drew on the *z* statistics providing the opportunity to examine whether the treatment effect of a (sub-)category was significantly (*p* < 0.05) different from zero. Forest plots were generated for the visualization of intervention effects. The interpretation of effect sizes followed the guidelines by Cohen [71]: small (SMD ≈ 0.20), medium (SMD ≈ 0.50), and large (SMD ≈ 0.80). If any single intervention showed a very large effect size with SMD > 3, we additionally performed the corresponding analysis without this sample to acquire information about the robustness of the result. Moreover, we conducted sensitivity analyses (via further subgroup analysis) to examine whether the results on the main category level differed between randomized and non-randomized controlled trials. Last, publication bias was explored on the basis of a visual inspection of funnel plots as deviations from funnel asymmetry can be interpreted as indicating a biased publication pattern [72]. All meta-analytic procedures were run with Review Manager, version 5.4.1 (Cochrane, London, UK).

## Results

### Summary of Studies (All Evaluation Studies)

**Fig. 1:**
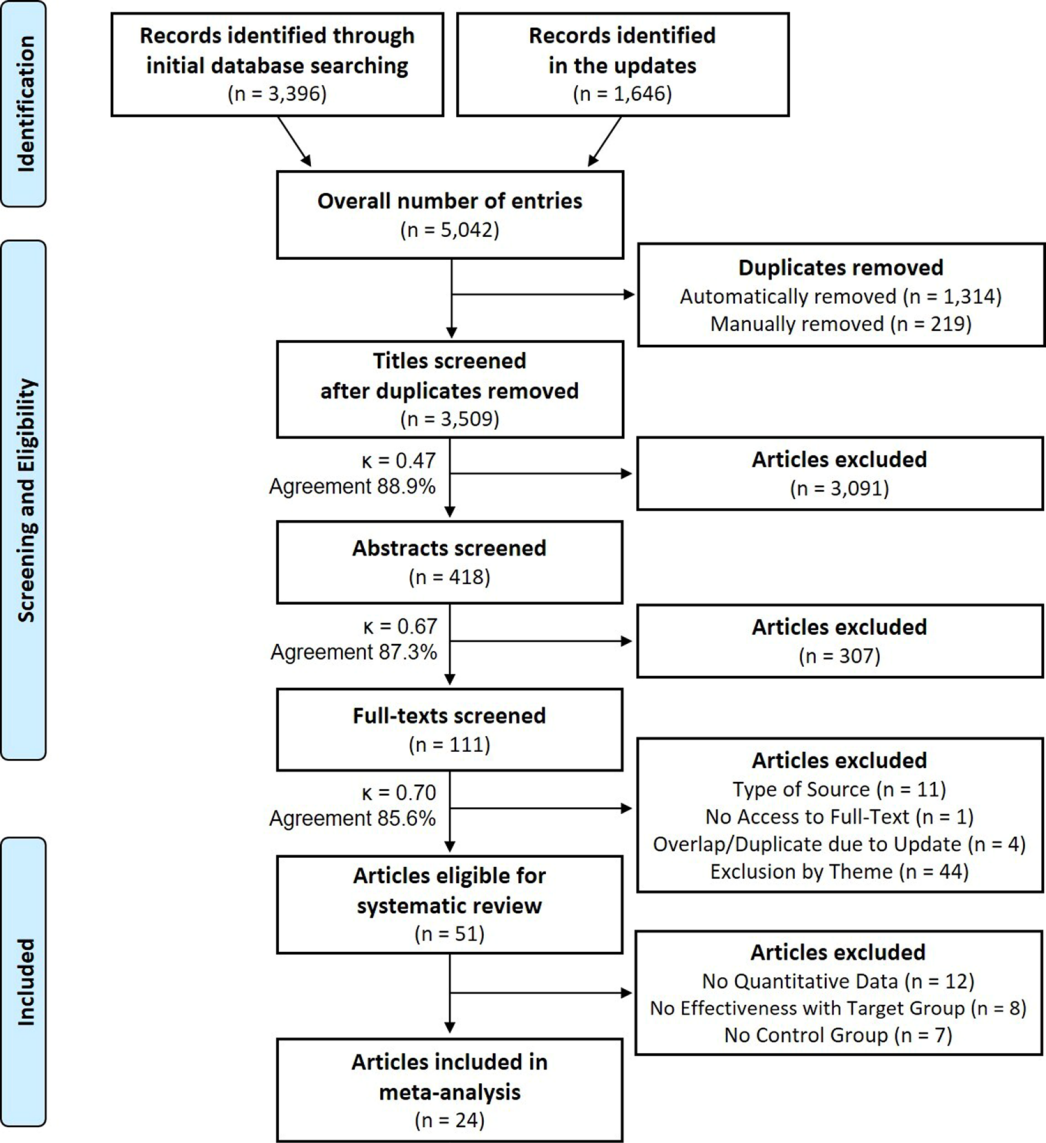
Preferred Reporting Items for Systematic Reviews and Meta-Analyses (PRISMA) flow diagram [53] showing the process of study identification and selection

We initially identified a total of 5042 potential articles. The multi-stage screening procedure resulted in *N* = 51 articles meeting the pre-defined eligibility criteria and, therefore, entering the systematic review (for a detailed visualization of the process, see Fig. 1). These 51 articles, in turn, reported evaluations of 48 separate PL interventions. An overview of the included studies and interventions is given in Tables 1 and 2.

**Table 1:** Summary of studies

Study*	Setting, country	Population	Design	Groups		Evaluation (quantitative vs qualitative)	Main findings
				IG	CG (if integrated)		
Alagul et al. (2012) [107]	Middle school in Ankara, Turkey	Pupils of the 7th grade	Case study with retrospective evaluation	Salsa dance unit: 40 min of physical moves plus 40 min of PL (*n* = 25)	–	Qualitative (subjective project evaluation, supported by results of a multiple-choice test)	Bloom taxonomy: after the lessons, pupils scored well in knowledge and analysis but low in synthesis
Arbour-Nicitopoulos et al. (2018) [108]; Weissman et al. (2021) [109]	Community recreation/soccer facilities in Ontario, Canada	Inactive children of grades 1–12 (14 m, 10 f)	Participatory case study with retrospective evaluation	Manual-based curriculum (QuickStart) and continuity (Give it a Try) program with child-centered games (Igniting Fitness Possibilities; *n* = 24)	–	Quantitative and qualitative (informal child, youth, and staff evaluations, questionnaires, log analysis)	24 children passed the QuickStart phase, 8 went on to the Give it a Try phase; overall positive evaluations, minor modifications suggested; coaches applied 36 different behavioral change techniques
Bremer et al. (2020) [80]*	After school programs in Hamilton, Canada	Children from after school (48 m, 42 f)	Cluster randomized controlled trial	Afterschool PL intervention (15 min of FMS stations + 15 min of active games; *n* = 47, age 9.1 ± 1.4 years)	Regular afterschool program (*n* = 43; age 10.5 ± 1.8 years)	Quantitative and qualitative (standardized questionnaires, open-ended feedback)	Positive effects on program leader perceptions, most outcomes in children were non-significant, implementation challenges
Campelo and Katz (2020) [101]	Community and independent- living centers in Calgary, Canada	Older adults (13 m, 27 f)	Randomized controlled trial	Exergame training in groups of five (Nintendo Wii-U, Wii Remote, Wii Balance Board; *n* = 15; mean age 72.6 years)	Two CGs: (1) conventional exercise (*n* = 14); (2) no training (*n* = 11)	Qualitative (focus groups)	Lack of familiarization with technology, positive perceptions about the implementation into exercise
Caput-Jogunica et al. (2009) [110]	Preschool children from kindergartens in Rijeka, Croatia	Preschool children aged 4–6 years (75 m, 61 f)	Within-subjects design (pre-post)	Extracurricular sports program with basic motor movements, games, track and field, gymnastics, dance, and aerobics (*n* = 136)	–	Quantitative (motor tests)	Significant improvements in six motor tests (attributable to strength, coordination, flexibility, and balance) over time
Choi et al. (2021) [90, 111, 112]*	One university in Hong Kong	University students (70% m)	Cluster randomized controlled trial	Student-centered higher PE program based on Sport Education (*n* = 188; age 18.53 ± 0.93 years)	PE classes with traditional teacher-directed methods (*n* = 184; age 18.57 ± 1.04 years)	Quantitative and qualitative (questionnaires, accelerometry, interviews, focus group, observations)	Increases in affective/social PL domains in both groups, greater increases in daily PA and greater reductions in disempowering climate in the IG 4 weeks after the intervention; lecturers cherish the value of the new concept but also perceive challenges
Clutterbuck et al. (2020) [78]	Outdoor community facilities in Queensland, Australia	Children with cerebral palsy aged 6–12 years (approximately 69% m)	Cluster randomized controlled trial (with waitlist)	Transition-focused sports training in soccer, netball, T-ball, and cricket (*n* = 39; age 9.0 ± 2.0 years)	Standard care with a log book (see study protocol; *n* = 25)	Quantitative and qualitative (session reports, physiotherapist and parent surveys)	Community as an adequate setting, high involvement; parents and physiotherapists described improvements in all PL domains; report of improvements in cognitive/physical domains
Collela and Bonasia (2019) [82]*	Primary schools in the south of Italy	First-grade children of a primary school (44 m, 40 f)	Randomized controlled trial	PE with a focus on the creativity, autonomy, and self-productivity of children (*n* = 40; age 6.88 ± 0.61 years)	Traditional PE curriculum (*n* = 44; age 7.02 ± 0.27 years)	Quantitative (motor test, questionnaire)	Significant improvements in the motor index and self-efficacy for male and female children in the IG but not in the CG
Countinho et al. (2018) [102]*	Two youth football clubs (regional level), Portugal	Attackers of m football teams (U15/U17)	Non-randomized controlled trial	PL and differential skills program for footballers (*n* = 15; age of U15: 14.2 ± 0.8 years, age of U17: 16.1 ± 0.7 years)	Regular football training (*n* = 15; age of U15: 13.9 ± 0.5 years, age of U17: 16.1 ± 0.7 years)	Quantitative (objective measures for technique, motor performance, creativity, and position)	Program showed improvements for the U15 in a range of variables across the four categories, less significant results for the U17
Coyne et al. (2019) [113]	Two elementary schools in southwestern Ontario, Canada	Elementary school children aged 8–12 years (154 m, 156 f)	Within-subjects design (pre-post)	FMS program with a focus on track and field inspired games (The Run Jump Throw Wheel Program; *n* = 310; age 10.5 ± 1.0 years)	–	Quantitative (questionnaire, heart rate monitoring)	Significant improvements in physical competence, knowledge/understanding, and overall PL but no changes in activity indicators; stronger effects in the suburban school
Crozier et al. (2021) [87]*	Afterschool community setting in Ottawa, Canada	Children from afterschool aged 5–12 years (13 m, 16 f)	Non-randomized controlled trial	Afterschool PA program facilitating a wide range of sports and athletic opportunities (ASAP; *n* = 14; age 8.25 ± 1.34 years)	Participants of another standard recreational afterschool program (HIGH FIVE; *n* = 15; age 8.63 ± 1.74 years)	Quantitative (anthropometrics, fitness and motor tests, questionnaires, accelerometry)	Between-group changes non-significant in all variables; given the small sample size, trends were found in BMI, aerobic capacity, and movement skills (in favor of the IG)
Demetriou et al. (2018) [114]*	Private primary schools in the south of Germany	Students of primary schools (101 m, 68 f; age 8.06 ± 1.21 years)	Non-randomized controlled trial	Primary school program with focus on (competitive) sports: daily PE lessons and additional PA bouts over the school day (*n* = 79)	Regular PE in a comparable primary school (*n* = 90)	Quantitative and qualitative (interviews and curriculum analysis, questionnaires)	Pupils of the sports-oriented schools showed higher values in standing long jump and PA attitudes; the sport focus of the school did not affect cognitive performance
Edwards et al. (2019) [115]	Medium-size primary schools in South Wales, UK	Grade five primary school children (aged 10–11 years)	Within-subjects design (pre-post)	Professional development program for primary school teachers to improve their knowledge and operationalization of PL, practical implementation for the target group (*n* = 68)	–	Qualitative (observations, collaborative discussion, interviews)	The PD program impacted teachers’ knowledge and operationalization of PL; embedding the principles of PL into PE resulted in sustainable changes in teachers’ practice
Everley (2021) [116]	Two schools in Mid Sussex, England, UK	Girls aged 5–12 years, rather inactive (25 f)	Case study with retrospective evaluation	Storytelling program for an enhancement of PL and a stronger engagement in PA (Active Literacy Through Storytelling; *n* = 25)	–	Qualitative (picture drawing, interview, group interview)	The program provided social opportunities for participants, ultimately developing their leadership and teamwork skills, and improved engagement
Farias et al. (2020) [117]	K-12 School in Northern Portugal	Grade seven school children aged 12–14 years (16 m, 10 f)	Participatory case study with retrospective evaluation	Year-long sport education curriculum (Sport Education) with action research elements (*n* = 26)	–	Qualitative (survey, interviews, focus group)	Students developed enthusiasm, competency, and literacy, which subsequently transformed their attitudes toward PE and PA
Gavigan et al. (2021) [118]	Primary schools in Ireland	Children from first or second class aged 6–10 years	Case study with retrospective evaluation	Skill-based multi-component school intervention (Moving Well-Being Well; *n* = 925; mean age 7.55 years)	–	Qualitative (focus groups, questionnaires)	The model and intervention increased FMS skills, confidence, enjoyment, and engagement, and improved behavior (focus in classroom)
George et al. (2016) [119]	After school activity center at Memorial University, Canada	Children in after school aged 6–12 years (7 m, 8 f)	Within-subjects design (pre-post)	Active video game sessions with sports and game content (Nintendo Wii Sport, Wii Sport Resort, Wii Play, Justn Dance 2; *n* = 15; age 7.9 ± 2.1 years)	–	Quantitative (questionnaires, activity and heart rate tracker, motor tests)	No general effect over time in any PL indicator; some single sex-specific improvements in facets of motivation and motor skill
Guerrero and Chandler (2018) [79]*	YMCA institutions in Ontario, Canada	Children aged 8–10 years (age 9.11 ± 0.60 years; 6 m, 3 f)	Non-randomized controlled trial	Regular soccer practice (Learn to Play Soccer program), complemented by imagery intervention (*n* = 5)	Regular basketball practice (Learn to Train Basketball program, *n* = 4)	Quantitative (motor tests, questionnaire)	Inconclusive results because of the sample size; anecdotal improvements in 6/9 variables for the IG, anecdotal evidence for better post-intervention values for the IG in 7/11 variables (via Bayesian H0/H1 comparisons)
Hassani et al. (2020) [81]*	Tehran province, Iran	Children with autism spectrum disorder (20 m, 10 f)	Randomized controlled trial	PL with motor skills program (ICPL; *n* = 11; age 8.55 ± 0.68 years)	Two CGs: (1) rest group (*n* = 9; age 8.70 ± 0.70); (2) FMS program (SPARK; *n* = 10, age 9.10 ± 0.87 years)	Quantitative (motor test battery)	The IG with the PL program showed significant better values in gross skill, fine skill, and motor skill after the intervention time compared with the CG and the SPARK group
Hassani et al. (2020) [83]*	Tehran province, Iran	Children with autism spectrum disorder (17 m, 13 f)	Randomized controlled trial	Program with a focus on FMS (ICPL; *n* = 15; age 9.13 ± 0.74 years)	No specific information (*n* = 15; age 9.26 ± 0.59 years)	Quantitative (fitness test, pedometer, questionnaires)	The IG improved significantly in all four PL indicators over time
Hassani et al. (2020) [75]	Provincial scale-up in British Columbia, Canada	Regional trainers delivering workshops for childcare providers	Within-subjects design [pre-post]	Capacity-building intervention (Appetite to Play) with a mediator approach: master (*n* = 2) and regional trainers (*n* = 88), local childcare providers (*n* = 2762)	–	Quantitative and qualitative (focus groups, interviews, open-ended questionnaires, website analysis)	Elements helpful to participants included availability of resources, equipment, sufficient space for activity, and an active play policy
Holler et al. (2019) [89]*	South east region of Austria	Physically inactive adults (19% m, 81% f)	Non-randomized controlled trial	Holistic physical exercise training with strength-, endurance, and multimodal activities (*n* = 31; age 44 ± 16 years)	No treatment (*n* = 30; age 45 ± 11 years)	Quantitative (questionnaire)	Significant improvements in PA, confidence, and PL for the IG; no changes in attitude, knowledge, and motivation or for the CG; stronger effects for persons with higher BMI
Holler et al. (2021) [84]*	Two different rural regions of Styria, Austria	Physically inactive adults (8% m, 92% f)	Non-randomized controlled trial	PL-based intervention with strength or mixed strength-endurance sessions (*n* = 33; age 53 ± 10 years)	No treatment (*n* = 22; age 50 ± 11 years)	Quantitative (questionnaire, fitness test)	Significant time-group interactions in PA behavior, knowledge, understanding, confidence, and the PL overall score in favor of the IG (only motivation non-significant); smaller effects for persons with better fitness
Invernizzi et al. (2019) [88]*	Primary schools in the province of Milan, Italy	Fifth-grade students (age 10.5 ± 0.5 years; 57 m, 64 f)	Cluster randomized controlled trial	PE program based on multi-teaching approaches (*n* = 62)	Standard PE lessons (*n* = 59)	Quantitative and qualitative (fitness/motor tests, questionnaires, interviews, lesson analysis)	Children in the IG reported higher satisfaction levels and stronger improvements in fitness/motor tests, enjoyment and PA levels relative to the CG
Invernizzi et al. (2021) [76]	Not fully determinable (“medium–high social context”)	Children without any swimming experience aged 5–6 years (age 5.9 ± 0.3 years; 47 m, 53 f)	Non-randomized controlled trial	Swimming course with a teacher-centered, linear pedagogical focus (*n* = 50)	– (comparison with other IG, see below)	Quantitative and qualitative (aquatic motor tests, questionnaire, pictorial scale)	The linear approach was more effective in promoting actual aquatic motor competence and better met parents’ expectations; the non-linear approach better reduced competence gaps and was more appreciated by the children
				Swimming course with a student-centered, non-linear pedagogical focus (*n* = 50)	– (comparison with other IG, see above)		
Johnstone et al. (2017) [85]*	Seven primary schools in Scotland, UK	Primary school children in grades 1–5 (90 m,106f)	Non-randomized controlled trial	School-based active outdoor play intervention with structured games and free play (Go2Play; *n* = 172; age 7.0 ± 1.1 years)	Usual PE class (*n* = 24; age 7.4 ± 109 years)	Quantitative (accelerometry, motor tests)	Significant time-group interactions in gross motor, locomotor, and school day PA indicators in favor of the IG; non-significant effects for object control skills
Kahlon et al. (2019) [120]	Ambulatory rehabilitation hospital, Canada	Children with cerebral palsy aged 8–17 years	Randomized controlled trial	FMS-based sport skill training program (BeFAST; *n* ≈ 10)	– (comparison with other IG, see below)	Qualitative (interviews)	The ability to practice movements of personal interest increased enjoyment; specific instruction of activities was an important factor in promoting skill development
				Lower limb strength training program (BeSTRONG; *n* ≈ 10)			
Kriellaars et al. (2019) [91]*	PE classes in Montreal, Canada	Grade 4 and 5 children aged 9–12 years (mean age 10.07 ± 0.77 years; 95 m, 116 f)	Non-randomized controlled trial	Student-centered circus arts instruction in PE (*n* = 110)	Standard PE instruction with traditional curriculum focusing on sports (*n* = 101)	Quantitative (motor tests, questionnaire)	Significant improvements in motor competence for both groups over time; most outcomes favored the IG, especially in grade 5; reduced the sex gap in motor skills in the IG
Kwan et al. (2019, 2020) [34, 92]*	Living learning community on the campus of a university in Canada	First-year university students (19 m, 46 f)	Non-randomized controlled trial	Manual-based movement skills program with games (PLUS Program; *n* = 39; age 17.85 ± 0.49 years)	No intervention (*n* = 26; age 17.85 ± 0.54 years)	Quantitative (motor skills, questionnaire, observation)	Significant time by condition effect for knowledge and understanding, motivation, and overall PL in favor of the IG; no interaction effect for movement competence and confidence
Lane et al. (2021) [77]	Community networks of schools and sport clubs in Canada	Parents (25 m, 8 f) of children aged 3–8 years	Within-subjects design (pre-post)	Group workshop and educational material for children’s parents (PLAYshop; *n* = 33; mean age 38.45 years)	–	Quantitative and qualitative (self-developed questionnaires, interviews)	All indicators of parents’ self-reported PL knowledge and confidence improved significantly; parents were satisfied with the workshop, considered it useful; parents and facilitators mentioned strengths and challenges
Lee et al. (2018) [121]	Four youth organization centers in Ottawa, Canada	Grade six children of youth organizations (90 m, 73 f)	Within-subjects design (pre-post)	Kids academy program in a community or summer camp format (*n* = 163; age 11.1 ± 0.8 years)	–	Quantitative and qualitative (questionnaires for children and parents)	Significant improvement in students’ knowledge scores; trend for parents indicating higher PA levels of their children after the IG; parents reported more discussions on health topics and better health behaviors of children at home
Lloyd (2016) [122]	Seven schools in Ottawa, Canada	Children from grades 1 to 9	Case study (phenomenological approach)	Adventure-based learning program with obstacle courses and vertical challenges (JungleSport Program; *n* = 153)	–	Qualitative (phenomenological observations, group interviews, journal entries)	The creative writing component encouraged expression, which promoted feelings of power and strength, translating to bodily awareness
Mandigo et al. (2019) [43]	Elementary school in southwestern Ontario, Canada	School children from grades 5 to 8 (6 m, 16 f)	Within subjects design (pre-post)	After-school program with Teaching Games for Understanding approach (PlaySport; *n* = 22)	–	Quantitative (rating of motor/fitness skills, questionnaires)	The participants improved significantly in four of 12 PL indicators (all from the fitness skills and participation domains); eight indicators did not change over time (e.g., all indicators of the movement/living skills domain)
Mateus et al. (2015) [103]*	PE institutions, Portugal	Male and female college students (age 20.4 ± 1.9 years)	Randomized controlled trial	Basketball program with differential learning and PL approaches (BasketCAL Program; *n* = 38)	Basketball tasks, not further specified (*n* = 38)	Quantitative (motor tests, video-assisted tactical assessment)	The IG showed stronger improvements in agility than the CG; no group differences in technical basketball skills; two of eight tactical indicators developed in favor of the IG, six remained non-significant
McLachlan et al. (2017 [73]	Childcare centers in low SES communities, New Zealand	Children aged 0–6 years	Non-randomized controlled trial (with waitlist)	Professional learning program for teachers (two centers) and PA sessions for children (Jumping Beans Program)	Wait control (two centers)	Quantitative and qualitative (questionnaire, interviews)	Some teachers made outdoor environmental changes, as well as focused more on FMS and promoting such skills; most teachers gained knowledge, skill, and confidence to promote PA
Pullen et al. (2020) [123]*	Three PE classes from a secondary school in Wales, UK	Boys and girls in grades 7–9 (20 m, 26 f)	Non-randomized controlled trial	Strength and conditioning program replacing regular PE (*n* = 23)	Regular PE classes (*n* = 23)	Quantitative (questionnaires, movement skills with rating)	Significant improvement in resistance training skills for the IG but not for the CG; no changes in jumping outcomes; increase in motivation only for the male IG
Santos et al. (2017) [124]*	Primary school in Portugal	Three and four graders of a primary school	Randomized controlled trial	Extracurricular sport activity program with a focus on creative thinking (Skills4Genius; *n* = 22; age 9.5 ± 0.7 years)	No specific information (*n* = 18; age 9.2 ± 0.4 years)	Quantitative (creative thinking drawing task, motor tests, motion/position analysis)	The intervention significantly promoted participants’ creative thinking, in-game creativity, and tactical performance; inconclusive effects on motor performance
Spencer et al. (2021) [125] (Study Protocol: Houser et al. (2019) [126]	Childcare centers in Nova Scotia, Canada	Preschoolers aged 3–5 years	Cluster randomized controlled trial	Seven-component loose parts material intervention (PLEY; *n* = 96)	Usual care (*n* = 87)	Quantitative (reported elsewhere) and qualitative (motor test, accelerometry, questionnaires, photo documentation, focus groups)	Educators’ perspective on a loose parts intervention component: loose parts are perceived positively but also as risky; importance of safety aspects, observations, and communication
Strobl et al. (2020) [127]*	Secondary schools in Bavaria, Germany	Pupils in grades 7–10 (105 m, 128 f)	Participatory research with a non-randomized controlled trial	Participatively developed, setting-specific measures for PE (*n* = 141)	Regular PE lessons (*n* = 92)	Quantitative and qualitative (protocols, questionnaire)	Knowledge and understanding differed significantly between intervention and control schools; large between-school effects, linkable to implementation conditions
Sum et al. (2020 [128]	Teacher education institution in Hong Kong	PE teachers (8 m, 1 f)	Within-subjects design (pre-post)	Continuing professional development program for PE teachers (PE-CPD; age 34.7 ± 8.28 years)	–	Qualitative (interviews, focus groups)	Teachers perceived the program improved their teaching and self-efficacy, as well as strengthened their confidence, competence, sense of self, and scientific knowledge of movement
Telford et al. (2020) [49]*	Seven primary schools (grade 5) in Australia	Grade 5 students (age 10.4 ± 0.4 years; approximately 49% m, 51% f)	Action research with a cluster randomized controlled trial	School development approach with peer coaching and mentoring for PE teachers (PEPL; *n* = 152)	Usual PE practice (*n* = 166; age 10.4 ± 0.4 years)	Student outcomes: quantitative and qualitative (accelerometry, motor test, questionnaires, focus groups, interviews)	The intervention had a positive effect on object control, no effect on PA indicators and enjoyment, a negative effect on physical competence; qualitative data suggest intervention enjoyment and higher motivation/confidence
Telford et al. (2020) [74]						Teacher/school outcomes: quantitative and qualitative (teacher observation, logbook analysis, interviews)	Greater lesson duration, instructional time, and PA volume in class; stronger PA school culture, staff supported the approach, higher teacher confidence for PE
Telford et al. (2021) [86]*	Childcare centers in New South Wales and southern Queensland, Australia	Preschool children aged 3–5 years (180 m, 134 f)	Cluster randomized controlled trial	Peer coach-based intervention for educators of childcare centers (AEL Intervention; *n* = 170; age 4.3 ± 0.4 years)	Usual practice (*n* = 144; age 4.3 ± 0.5 years)	Quantitative and qualitative (accelerometry, logbooks)	The peer coach realized 164 of 176 planned site visits; children in the IG schools spent significantly more time with PA (moderate to vigorous: + 16 min/day; total: + 28 min/day) than those in the CG schools
Wainwright et al. (2020) [129]*	Ten primary schools in Wales, UK	Pupils in primary school (age 5.53 ± 0.62 years)	Non-randomized controlled trial	Multi-component whole school intervention with six key features (SKIP-Cymru; *n* = 134)	Usual practice (*n* = 21)	Quantitative and qualitative (motor tests, interviews)	Stronger improvement in motor competence for the IG; students were more engaged, motivated, and improved their gross motor skills; staff developed confidence and an understanding of their practices; parent engagement seemed to promote children’s enjoyment and confidence
Warner et al. (2020) [130]	SFD facility in Toronto, Canada	Children of a SFD facility aged 6–10 years (23 m, 22 f)	Within-subjects design (pre-post)	Community-based SFD program with a focus on FMS and games (*n* = 45; age 7.93 ± 1.37 years)	–	Quantitative (motor tests, questionnaires)	Significant improvements in self-perceptions of PL and FMS (especially for running and balance), strongest impact on low baseline performers
Wayne (2018) [131]	Early childhood centers and NRL clubs from Queensland, Australia	Children aged 3–5 years (65% m, 35% f)	Case study with retrospective evaluation	Childhood development program with a multifaceted learning approach within a NRL initiative (Munchkin League Program)	–	Qualitative (interviews with parents and staff)	Program increased enjoyment, confidence, and engagement, and promoted a sense of achievement; participants’ physical skills, running, motor development, body movement, and coordination improved as well
Wright et al. (2020) [132]*	PE classes in elementary schools, Canada	Children aged 4–7 (295 m, 253 f)	Non-randomized controlled trial	Job-embedded professional development for generalist teachers and implementation in PE (*n* = 283; children mean age 7.9 ± 1.7 years)	Usual practice without teacher development (*n* = 268; children mean age 7.6 ± 1.6 years)	Quantitative and qualitative (questionnaires and interviews for teachers, motor skill rating)	Teachers showed high program satisfaction, good observability of satisfaction; increases in confidence and intentions to deliver PL in PE; access to online resources was beneficial; change in children’s motor skills did not differ between groups (except in throwing)

*ASAP* Afterschool Activity Programs, *BMI* body mass index, *CG* Control Group, *f* female, *FMS* fundamental movement skills, *IG* Intervention Group, *m* male, *NRL* National Rugby League, *PA* physical activity, *PD* Professional Development, *PE* physical education, *PL* physical literacy, *SES* socioeconomic status, *SFD* Sport for Development, *YMCA* Young Men’s Christian Association

^*^Articles with an asterisk entered the meta-analysis; information on sex is reported in the column “population”, while age characteristics are included in the columns of both “groups” (only if available for both groups)

**Table 2:** Summary of the theoretical background, design, and content of PL interventions

Study*	Theoretical foundation of the intervention				Delivery characteristics (intervention with the target group)					
	Holistic PL definition	Degree of foundation in PL theory^a^	PL domains addressed^b^	Consideration of intertwining of PL domains	Length	Frequency	Duration	Intensity	Intervention approach^c^	Intervention training
Alagul et al. (2012) [107]	No	Theory inspired	PC, K&U	No	4 weeks	1 × /week	80 min	N/A	Direct	Not mentioned
Arbour-Nicitopoulos et al. (2018) [108], Weissman et al. (2021) [109]	Yes	Theory inspired	PC*, K&U, M&C	Yes	Depending on group: 16–28 weeks	Depending on group: 1–2 × /week	90 min	N/A	Direct	Yes
Bremer et al. (2020) [80]*	Yes	Theory based	PC, K&U, M&C	Yes	12 weeks	5 × /week	30 min	N/A	Direct	Yes
Campelo and Katz (2020) [101]	Yes	Intervention not sufficiently related to PL theory	PC*	No	6 weeks	3 × /week	N/A	N/A	Direct	Not mentioned
Caput-Jogunica et al. (2009) [110]	No	Intervention not sufficiently related to PL theory	PC*	No	9 months	4 × /week	45 min	N/A	Direct	Not mentioned
Choi et al. (2021, 2021, 2021) [90, 111, 112]	Yes	Theory inspired	PC, K&U, M&C	Yes	10 weeks	1 × /week	90 min	11.5% vigorous; 27.3% walking	Indirect	Yes
Clutterbuck et al. (2020) [78]	Yes	Theory inspired	PC, K&U, M&C	No	8 weeks	1 × /week	1 h	N/A	Direct	Yes
Collela and Bonasia (2019) [82]*	Yes	Theory inspired	PC, K&U, M&C	Yes	Approximately 6 months	N/A	N/A	N/A	Direct	Not mentioned
Countinho et al. (2018) [102]*	No	Theory based	PC*	No	10 weeks	2 × /week	25 min (integrated)	N/A	Direct	Not mentioned
Coyne et al. (2019) [113]	Yes	Theory inspired	PC*	No	10 weeks	2 × /week	40 min	23.5% light PA, 49.6% moderate PA, 27.3% vigorous PA	Direct	Yes
Crozier et al. (2021) [87]*	Yes	Theory inspired	PC*	No	Approximately 6 months	5 × /week	3 h	N/A	Direct	Yes
Demetriou et al. (2018) [114]*	Yes	Intervention not sufficiently related to PL theory	PC*, M&C	No	6 months	5 × /week	90 min	N/A	Direct	Not mentioned
Edwards et al. (2019) [115]	Yes	Theory inspired	PC, K&U, M&C	No	6 months	2 × /week	60 min	N/A	Indirect	Yes
Everley, 2021 [116]	Yes	Theory inspired	K&U, M&C	No	N/A	N/A	N/A	N/A	Direct	Yes
Farias et al. (2020) [117]	Yes	Theory inspired	PC, K&U, M&C	Yes	1 school year (approximately 10 months)	2 × /week	45 min/90 min	N/A	Direct	Not mentioned
Gavigan et al. (2021) [118]	Yes	Theory inspired	PC*, M&C	Yes	8 weeks	2 × /week (PE class) + daily (classroom) 1 × /week (home)	30 min (PE class) + 5–10 min (classroom)	N/A	Direct	Yes
George et al. (2016) [119]	No	Intervention not sufficiently related to PL theory	Not explicitly mentioned	No	6 weeks	2 × /week	At least 20 min	Average heart rate 113.5/114.9 bpm; peak heart rate 150.1/156.5 bpm	Direct	Not mentioned
Guerrero and Chandler, 2018 [79]*	Yes	Intervention not sufficiently related to PL theory	PC, K&U, P&C	No	4 weeks	3 × /week (1 × with researcher, 2 × alone)	Imagery: approximately 10 min more than regular training	N/A	Direct	Not mentioned
Hassani et al. (2020) [81]*	Yes	Theory inspired	PC*, K&U	No	3 months (16 sessions overall)	2 × /week	60 min	N/A	Direct	Not mentioned
Hassani et al. (2020) [83]*	Yes	Theory inspired	PC*, K&U	No	2 months (16 sessions overall)	2 × /week	80 min	N/A	Direct	Not mentioned
Hassani et al. (2020 [75]	No	Theory inspired	–	No	Once		Depending on modality: 1.5–3 h	N/A	Indirect	Yes
Holler et al. (2019) [89]*	Yes	Theory based	PC, K&U, M&C	Yes	15 weeks	1 × /week (with participants able to attend 1, 2 or 3 sessions on the day)	50 min	%HFmax pred 67 ± 9; Borg 12 ± 2	Direct	Not mentioned
Holler et al. (2021) [84]*	Yes	Theory based	PC, K&U, M&C	Yes	14 weeks	1 × /week (with participants able to attend 1 or 2 sessions on the day)	50 min	%HFmax 67–76; Borg 12–13	Direct	Not mentioned
Invernizzi et al. (2019) [88]*	Yes	Theory inspired	PC, K&U, M&C	Yes	12 weeks	2 h/week		N/A	Direct	Yes
Invernizzi et al. (2021) [76]	Yes	Theory inspired	PC*	Yes	15 weeks	2 × /week	50 min	N/A	Direct	Not mentioned
	Yes	Theory inspired	PC, M&C	Yes	15 weeks	2 × /week	50 min	N/A	Direct	Not mentioned
Johnstone et al. (2017 [85]*	No	Theory based	PC*	No	5 months	4 classes: 2 × /week; 7 classes: 1 × /week	60 min	50.8% light PA and 30.1% MVPA	Direct	Yes
Kahlon et al. (2019) [120]	Yes	Theory based	PC*	No	6 weeks	2–3 × /week (active) + 2–3 × /week (home)	45 min (active) + 3–5 min (home)	N/A	Direct	Yes
	Yes	Theory based	PC*	No	6 weeks	2–3 × /week (active) + 2–3 × /week (home)	45 min (active) + 3–5 min (home)	N/A	Direct	Yes
Kriellaars et al. (2019) [91]*	Yes	Theory based	PC, K&U, M&C	Yes	N/A	3 × /week	50–60 min	N/A	Direct	Yes
Kwan et al. (2019, 2020 [34, 92]*	Yes	Theory based	PC, K&U, M&C	No	11 weeks	1 × /week	60 min	N/A	Direct	Yes
Lane et al. (2021 [77]	Yes	Theory based	PC, K&U, M&C	Yes	Once		75 min	N/A	Direct	Yes
Lee et al. (2018) [121]	Yes	Theory based	PC, K&U*, M&C	No	4 weeks or 1 week (community or camp format)	2 × /week or 2 × /day (community or camp format)	90 min	N/A	Direct	Yes
Lloyd (2016) [122]	Yes	Theory inspired	–	No	3–5 days	N/A	N/A	N/A	Indirect	Not mentioned
Mandigo et al. (2019) [43]	Yes	Theory inspired	PC, K&U	Yes	8 weeks	Approximately 3 × /week	Approximately 60 min	N/A	Direct	Yes
Mateus et al. (2015) [103]*	No	Theory inspired	PC*, K&U	No	8 weeks	2 × /week	120 min	N/A	Direct	Not mentioned
McLachlan et al. (2017 [73]	No	Theory based	PC*, M&C	No	10 weeks	1 × /week	45 min	N/A	Indirect	Yes
Pullen et al. (2020) [123]*	No	Theory inspired	PC*, M&C	Yes	6 weeks	1.5 × /week	N/A	N/A	Direct	Not mentioned
Santos et al. (2017) [124]*	No	Theory inspired	PC*, K&U	Yes	5 months (total: 60 sessions)	3 × /week	60 min	N/A	Direct	Not mentioned
Spencer et al. (2021) [125] , study protocol: Houser et al. (2019) [126])	Yes	Theory inspired	–	No	6–8 months	N/A	N/A	N/A	Indirect	Yes
Strobl et al. (2020) [127]*	Yes	Theory inspired	PC, K&U*, M&C	Yes	1 school year	N/A	N/A	N/A	Indirect	Yes
Sum et al. (2020) [128]	Yes	Theory inspired	PC	No	3 months (16 h overall)	N/A	N/A	N/A	Direct	Not mentioned
Telford et al. (2020) [49]*	Yes	Theory inspired	PC, K&U	No	33 weeks	Different measures, including one additional PE lesson/week and four activity sessions (15–40 min) in the school yard		N/A	Indirect	Yes
Telford et al. (2020) [74]	Yes	Theory inspired	PC, K&U, M&C	Yes	22 weeks	Components daily integrable (no detailed information)	5–30 min for each component	N/A	Indirect	Yes
Telford et al. (2021) [86]*	Yes	Theory inspired	PC	No	8 weeks	2 × /week	45 min	N/A	Indirect	Yes
Wainwright et al. (2020) [129]*	Yes	Theory based	PC, K&U, M&C	Yes	9 days	Daily	200–285 min PA/day	N/A	Direct	Not mentioned
Warner et al. (2020) [130]	Yes	Theory inspired	PC	No	8 sessions		45 min	N/A	Direct	Not mentioned
Wayne (2018) [131]	Yes	Theory based	PC, K&U, M&C	Yes	N/A	N/A	N/A	N/A	Indirect	Yes
Wright et al. (2020) [132]	Yes	Theory based	PC, K&U, M&C	Yes	N/A	N/A	N/A	N/A	Indirect	Yes

A detailed description of the studies can be retrieved from the original review of the design and content of PL interventions [29]; *articles with an asterisk entered the meta-analysis

*HFmax* maximum heart frequency, *K&U* knowledge and understanding, *M&C* motivation and confidence, *MVPA* moderate-to-vigorous physical activity, *PC* physical competence, *PE* physical education, *PL* physical literacy, *pred* predicted

^a^Using the theory coding scheme [64], it was analyzed whether PL theory just loosely dictated intervention content (theory inspired) or whether intervention components were directly derived from PL theory, ideally with perceivable theory-content links (theory based); the agreement between both raters for this categorization as part of the full text-based extraction process was 83.8%

^b^In studies marked with an asterisk (*), researchers placed particular emphasis on a certain PL domain; consider that the domains were assessed differently (e.g., objective motor tests vs subjective evaluations for the level of physical competence, see also Table 1)

^c^A *direct* intervention approach intervenes directly at the target group level or lists a prescribed program content under control of the researchers; in contrast, an *indirect* intervention approach intervenes at the level of mediators (e.g., teacher education [115] or train-the-trainer approach [75]) to reach a target group without detailed/prescribed content

A total of 12 interventions concentrated exclusively on qualitative *evaluation data* (25.0%), while the evaluation of 19 interventions relied completely on quantitative data (39.6%); 17 interventions drew on both data modalities (35.4%) and can, therefore, be characterized as mixed-methods or multi-method evaluation efforts. When analyzing the 48 interventions by *study design*, 15 interventions applied a randomized controlled study design (31.3%), 16 interventions applied a non-randomized controlled study design (33.3%), ten interventions applied a (non-controlled) pre-post study design (20.8%), and seven interventions applied a retrospective case study design (14.6%).

### Study Quality and Domains Addressed (Quantitative Studies)

Among the 36 interventions (reported in 39 articles) providing any quantitative data, 26 included at least one outcome attributable to physical competence (72.2%). Moreover, 17 interventions listed at least one outcome related to motivation and confidence (47.2%), 15 interventions at least one outcome related to PA behavior (41.7%), and 12 interventions at least one outcome related to knowledge and understanding (33.3%). A total of nine interventions reported an overall score of PL, representing an aggregate value across the different domains (25.0%). Last, ten studies encompassed outcomes from other persons (27.8%), such as teachers (e.g., [73, 74]), educators (e.g., [75]), parents (e.g., [76, 77]), and physiotherapists (e.g., [78]).

Aggregated information regarding the study quality of the 36 interventions with a quantitative focus can be retrieved from Table 3. The methodological criteria of blinding and of whether data were adequately addressed were met least frequently (13.9%). Importantly, if blinding was fulfilled, this quality only referred to the concealed allocation process and not to the actual intervention delivery. Obviously, the application of imputation procedures or intention-to-treat paradigms cannot be declared as a standard of the research field. We also found that randomizations (30.6%) and reliable measurements (55.6%) are not widely established within the PL interventions literature. Most studies performed measurements before and after the interventional period (94.4%).

**Table 3:** Methodological quality of the 36 interventions that provided any quantitative data

Item	Criterion fulfilled	Criterion not fulfilled or not sufficient information given
(1) Availability of control group	27 (75.0%)	9 (25.0%)
(2) Randomization	11 (30.6%)	25 (69.4%)
(3) Multiple measurement of the outcome (both pre and post)	34 (94.4%)	2 (5.6%)
(4) Blinding	5 (13.9%)	31 (86.1%)
(5) Description of the completeness of outcome data (e.g., dropout or attrition rate)	27 (75.0%)	9 (25.0%)
(6) Incomplete data adequately addressed (e.g., imputation techniques, intention to treat)	5 (13.9%)	31 (86.1%)
(7) Free of suggestion of selective outcome reporting	19 (52.8%)	17 (47.2%)
(8) Outcomes measured reliably (in a quantitative sense)	20 (55.6%)	16 (44.4%)

### Effectiveness (Meta-Analysis)

As 23 interventions (described in different 24 articles) reported quantitative outcomes of their end users and simultaneously included a control group (i.e., controlled design), these data were used for a meta-analytical investigation. These interventions reported the results of a maximum of 1622 participants in the intervention groups and of 1369 participants in the control groups.

#### Sensitivity Analysis and Publication Bias

We did not find any hints that the *randomization criterion* as an indicator of study quality (see Table 5 of the ESM) meaningfully influenced the effect size level of physical competence (*χ*^2^(1) = 1.00, *p* = 0.32; *I*^2^ = 0.2%). Similar results were found for the main categories of motivation and confidence (*χ*^2^(1) = 0.03, *p* = 0.87; *I*^2^ = 0%) as well as PA behavior (*χ*^2^(1) = 0.01, *p* = 0.92; *I*^2^ = 0%). Because of the limited number of studies for each subcategory (*k* < 3), the sensitivity analyses could not be performed or explored for the main categories of knowledge and understanding as well as the total PL score. In contrast, the visual inspection of funnel plots suggested that the literature of PL interventions may be systematically affected by *publication bias* (see Fig. 1 of the ESM). On the main category level, asymmetry patterns could be identified for the physical competence and the motivation and confidence domains. Because of the limited number of studies, such a pattern could only be vaguely assumed for the remaining three subcategories. When plotting the outcomes of all main categories into one combined chart, there was a clear tendency toward selective publication.

#### Physical Competence

**Fig. 2:**
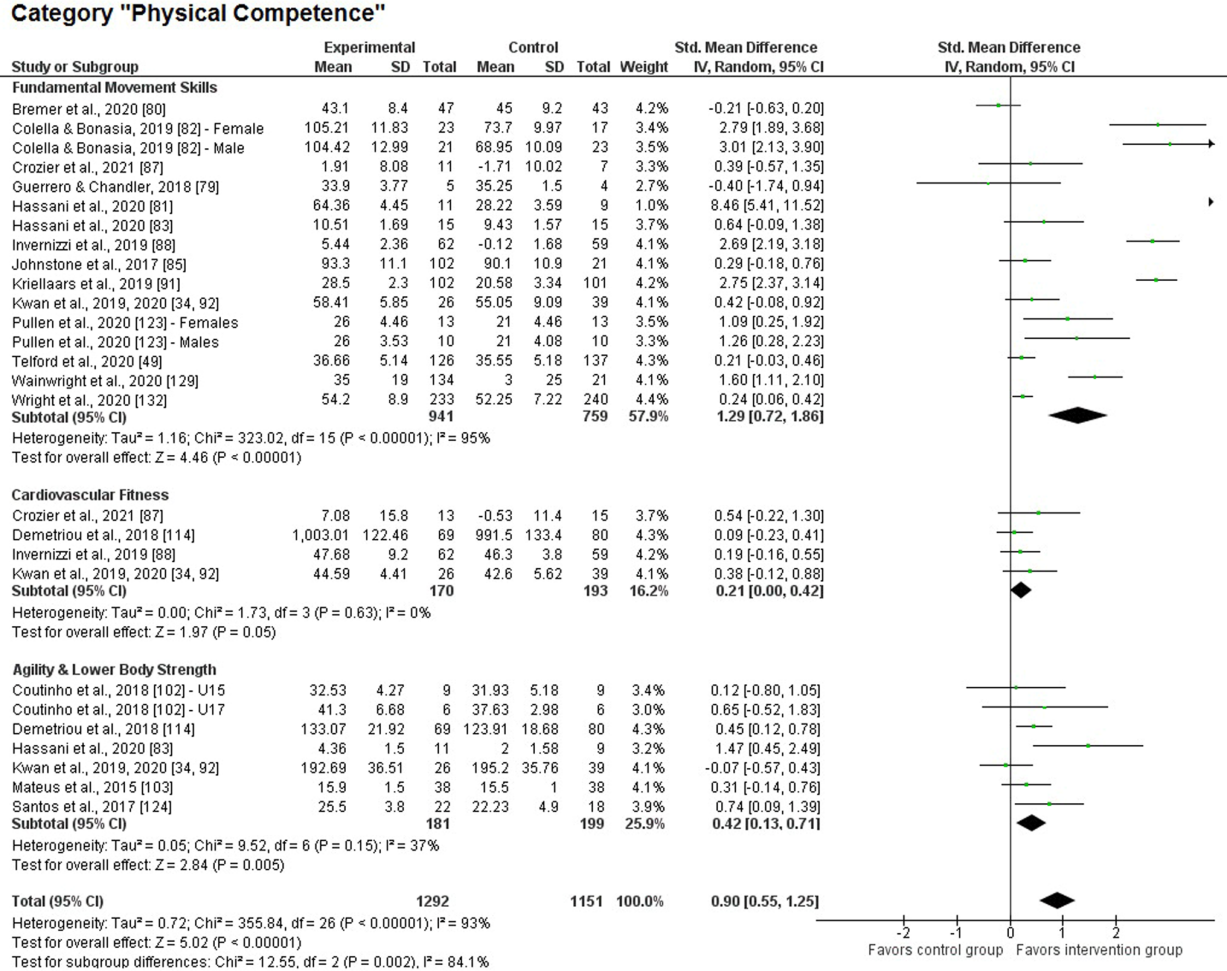
Forest plot of the main category “physical competence” and their corresponding sub-categories. Note: as mentioned in Sect. 2.5, effects were adjusted for intervention studies showing significant differences at baseline or listing several indicators per category. Against this background, some values displayed for the control group do not always correspond with the values reported in the original articles (for details, see Table 4 of the ESM). *CI* confidence interval, *df* degrees of freedom, *IV* inverse variance, *SD* standard deviation

Interventions that drew on PL either as a theoretical underpinning or as an evaluation outcome had a significant impact on indicators of physical competence (see Fig. 2), showing a large effect size overall (*k* = 21; *z* = 5.02, *p* < 0.001; SMD 0.90, 95% CI 0.55–1.25). The heterogeneity was high in this main category (*χ*^2^(26) = 355, *p* < 0.001; *I*^2^ = 93%).

We detected a significant large-size effect for the subcategory of *fundamental movement skills* (*k* = 16; *z* = 4.46, *p* < 0.001; SMD 1.29; 95% CI 0.72–1.86). Among the 16 samples included, two descriptively favored the control group [79, 80]. For this subcategory, we registered substantial heterogeneity (*χ*^2^(15) = 323, *p* < 0.001; *I*^2^ = 95%) and found two datasets with extreme (SMD > 3) effect sizes [81, 82]. If these two interventions were considered as outliers and excluded within the scope of a separate robustness analysis, we still found a large and significant treatment effect for this subcategory (*k* = 14; *z* = 3.52, *p* < 0.001; SMD 0.99, 95% CI 0.44–1.55). In contrast, the four interventions of the *cardiorespiratory fitness* subcategory must all be characterized as non-significant endeavors. Accordingly, we recorded only a small, marginally significant effect for this subcategory (*k* = 4; *z* = 1.97, *p* = 0.05; SMD 0.21, 95% CI 0.00–0.42), although heterogeneity was negligible (*χ*^2^(3) = 1.73, *p* = 0.63; *I*^2^ = 0%). Finally, the interventions exerted positive effects on parameters attributable to the *agility and lower body strength* subcategory (*k* = 7; *z* = 2.84, *p* = 0.005; SMD 0.42, 95% CI 0.13–0.71), with four out of seven studies exhibiting a significant effect. Nevertheless, the heterogeneity was meaningful for this subcategory (*χ*^2^(6) = 9.52, *p* = 0.15; *I*^2^ = 37%). From an inferential statistic perspective, the significant heterogeneity between the three subcategories implicated different effects of PL interventions depending on the outcome (*χ*^2^(2) = 12.6, *p* = 0.002; *I*^2^ = 84%). The effects on fundamental movement skills were higher than the effects on cardiorespiratory fitness (*χ*^2^(1) = 12.4, *p* < 0.001; *I*^2^ = 92%) as well as agility and lower body strength (*χ*^2^(1) = 7.25, *p* = 0.007; *I*^2^ = 86%). The comparison of the cardiorespiratory fitness with the agility and lower body strength subcategory was non-significant (*χ*^2^(1) = 1.33, *p* = 0.25; *I*^2^ = 25%).

#### Motivation and Confidence

**Fig. 3:**
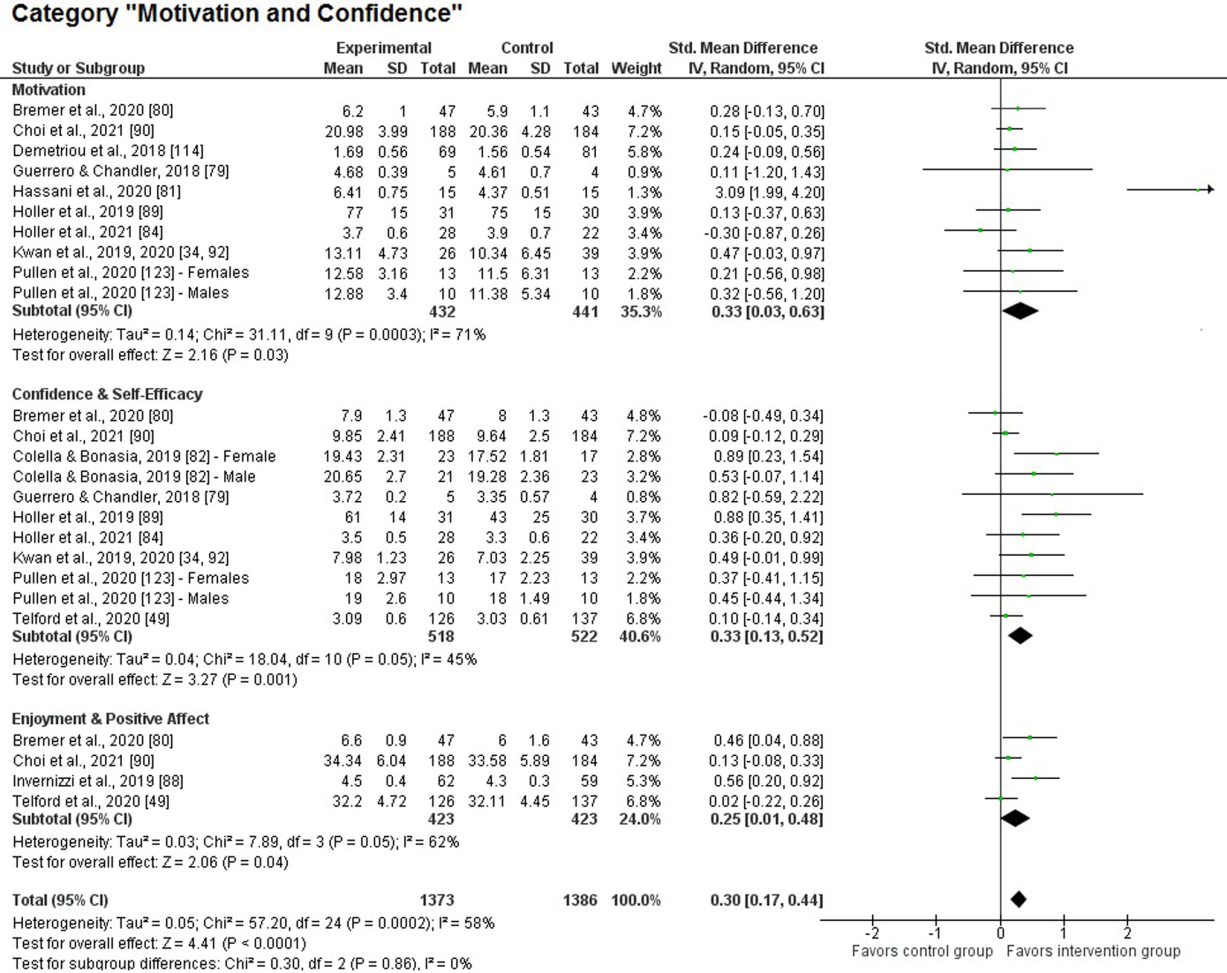
Forest plot of the main category “motivation and confidence” and their corresponding sub-categories. Note: as mentioned in Sect. 2.5, effects were adjusted for intervention studies showing significant differences at baseline or listing several indicators per category. Against this background, some values displayed for the control group do not always correspond with the values reported in the original articles (for details, see Table 4 of the ESM). *CI* confidence interval, *df* degrees of freedom, *IV* inverse variance, *SD* standard deviation

We identified a significant positive treatment effect by the interventions for the motivation and confidence domain (*k* = 14; *z* = 4.41, *p* < 0.001; SMD 0.30, 95% CI 0.17–0.44), accompanied by meaningful heterogeneity (*χ*^2^(24) = 57.2, *p* < 0.001; *I*^2^ = 58%). When analyzing the subcategory of *motivation* (see Fig. 3)*,* only one of the ten samples demonstrated a significant difference between groups after the intervention [83] and there was even one study in the negative effect range [84]. However, we detected a significant overall impact of low-to-moderate size (*k* = 10; *z* = 2.16, *p* = 0.03; SMD 0.33, 95% CI 0.03–0.63). The analysis suggested significant and substantial heterogeneity for this subcategory (*χ*^2^(9) = 31.1, *p* < 0.001; *I*^2^ = 71%). However, as the study with the significant result revealed an extreme treatment effect (SMD 3.09), we additionally ran the meta-analytical procedure without this intervention, leading to a marginalization of heterogeneity (*χ*^2^(8) = 4.72, *p* = 0.79; *I*^2^ = 0%). Despite a reduction in the absolute value, the significant effect with the nine samples remained (*k* = 9; *z* = 2.60, *p* = 0.009; SMD 0.18, 95% CI 0.04–0.32). Eleven studies provided an assessment of *confidence and self-efficacy* but only two intervention samples displayed a significant effect. In addition, one study showed higher post-test values for the control group [80]. Nevertheless, for this subcategory, we registered a significant effect of low-to-moderate magnitude (*k* = 11; *z* = 3.27, *p* = 0.001; SMD 0.33, 95% CI 0.13–0.52) with meaningful heterogeneity (*χ*^2^(10) = 18.0, *p* = 0.05; *I*^2^ = 45%). The exploratory analyses with *enjoyment and positive affect* as a subcategory surpassed the significance level (*k* = 4; *z* = 2.06, *p* = 0.04; SMD 0.25, 95% CI 0.01–0.48) and demonstrated meaningful heterogeneity (*χ*^2^(3) = 7.89, *p* = 0.05; *I*^2^ = 62%). Taken together, the three subcategories of this domain did not differ statistically with regard to their effect size (*χ*^2^(2) = 0.30, *p* = 0.86; *I*^2^ = 0%).

#### Knowledge and Understanding

We found a significant medium-size treatment effect for the domain of knowledge and understanding (*k* = 7; *z* = 4.31, *p* < 0.001; SMD 0.54, 95% CI 0.30–0.79). Concurrently, the heterogeneity was both significant and meaningful for this main category (*χ*^2^(9) = 34.1, *p* < 0.01; *I*^2^ = 74%).

**Fig. 4:**
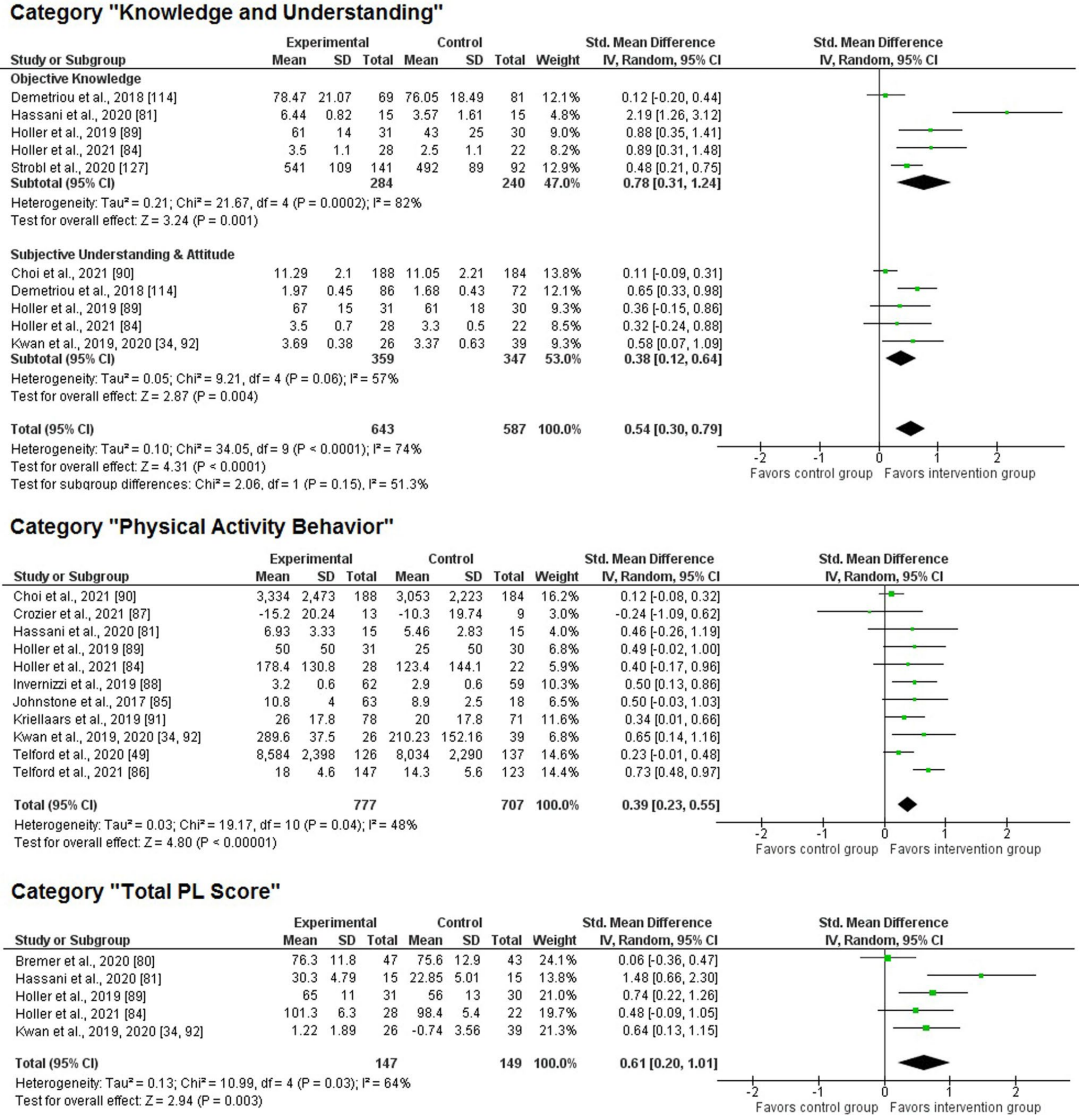
Forest plot of the main categories “knowledge and understanding” (including their corresponding sub-categories), “physical activity behavior”, and “total PL score”. Note: as mentioned in Sect. 2.5, effects were adjusted for intervention studies showing significant differences at baseline or listing several indicators per category. Against this background, some values displayed for the control group do not always correspond with the values reported in the original articles (for details, see Table 4 of the ESM). *CI* confidence interval, *df* degrees of freedom, *IV* inverse variance, *PL* physical literacy, *SD* standard deviation

Upon closer examination at the subcategory level (see Fig. 4), five intervention studies targeted *objective knowledge* through the application of a test with correct and incorrect answers. Four of these interventions entailed significant treatment effects, resulting in a significant overall effect of moderate-to-large size (*k* = 5; *z* = 3.24, *p* = 0.001; SMD 0.78, 95% CI 0.31–1.24). In addition, we registered significant and meaningful heterogeneity for this subcategory (*χ*^2^(4) = 21.7, *p* < 0.001; *I*^2^ = 82%). Five interventions included an operationalization of *subjective understanding or attitude*; also for this subcategory, we identified a significant impact of low-to-moderate size (*k* = 5; *z* = 2.87, *p* = 0.004; SMD 0.38, 95% CI 0.12–0.64) but substantial heterogeneity as well (*χ*^2^(4) = 9.21, *p* = 0.06; *I*^2^ = 57%). Albeit not significant, the magnitude of the effects differed meaningfully between both subcategories (*χ*^2^(1) = 2.06, *p* = 0.15; *I*^2^ = 51%).

#### PA Behavior

A total of 11 interventions employed PA assessments after the intervention. While six interventions used accelerometers [49, 85–88] or pedometers [83] as objective measurements, five interventions drew on subjective self-reports via questionnaires [34, 84, 89–92]. Apart from one study (SMD − 0.24 [87]), the mean values of all endeavors were located in the positive area by favoring participants in the intervention groups. The meta-analysis revealed that PL interventions had a significant, positive impact on individuals’ PA behavior of low-to-moderate size (*k* = 11; *z* = 4.80, *p* < 0.001; SMD 0.39, 95% CI 0.23–0.55). Significant and meaningful heterogeneity was recorded for this main category (*χ*^2^(10) = 19.2, *p* = 0.04; *I*^2^ = 48%).

#### Total PL

Finally, five interventions contained a multidimensional score of PL (21.7%). The scoring algorithm varied among the respective studies, from z-scores across three PL domains [34, 92], sum scores across four domains/subscales [83, 84], to five subscales [89]. In three of the five studies, the corresponding intervention content entailed significant treatment effects. The meta-analytic techniques indicated a significant effect for this main category, moderate to high in magnitude (*k* = 5; *z* = 2.94, *p* = 0.003; SMD 0.61, 95% CI 0.20–1.01). The heterogeneity across the five studies with a PL score was both significant and substantial (*χ*^2^(4) = 11.0, *p* = 0.03; *I*^2^ = 64%).

#### Statistical Comparison between the Main Categories

Combined, the calculations displayed (as reported) significant treatment effects for all five outcome categories. However, an analysis with all categories together suggests that the PL interventions exerted differential effects on the outcome categories (*χ*^2^(4) = 12.0, *p* = 0.02; *I*^2^ = 67%). Descriptively, the included interventions achieved the highest impact on the physical competence domain (SMD 0.90) and the lowest impact on the motivation and confidence domain (SMD 0.30). When direct comparisons between single categories are undertaken, the effect size of the aggregated physical competence domain differed significantly from the domains of motivation and confidence (*χ*^2^(1) = 9.69, *p* = 0.002; *I*^2^ = 90%) as well as PA (*χ*^2^(1) = 6.58, *p* = 0.01; *I*^2^ = 85%). Furthermore, we recorded substantial heterogeneity when contrasting the effect of the motivation and confidence category with the effects of the knowledge and understanding (*χ*^2^(1) = 2.83, *p* = 0.09; *I*^2^ = 65%) and the PL score (*χ*^2^(1) = 1.96, *p* = 0.16; *I*^2^ = 49%) categories. Meaningful heterogeneity was also found between the effects of the PA and the PL score category (χ^2^(1) = 2.63, *p* = 0.11; I^2^ = 62%).

## Discussion

With its holistic view, the concept of PL has inspired both research and practice in comprehensively targeting PA over the globe. The goal of this systematic review with a meta-analysis (building on previous analyses [29]) was to provide a broad overview of evaluation studies that refer to PL as a theoretical underpinning or as an explicit outcome and, subsequently, to examine their quantitative effectiveness. In summary, the present study uncovered a wide range of evaluative approaches from qualitative studies, on the one hand, to quantitative studies, on the other hand. In this regard, the field of PL interventions demonstrates methodological openness by not generally favoring a certain paradigmatic direction. In their review of definitions, Edwards et al. [16] highlighted the benefit of diversity in the area of PL, simultaneously calling for a culture of transparency and tolerance with different approaches. Underpinned by the rigorous approach of searching and selecting intervention studies, the current overview reflected that also the more applied field of interventions follows this idea by cultivating both qualitative-phenomenological and quantitative-nomothetic approaches (or a combination).

However, the more detailed analysis of the quantitative evaluation approaches revealed that the holistic character of PL did not sufficiently reach the evaluation level of interventions. Only three intervention projects included separate outcomes of all PL domains [34, 83, 92] or reported an aggregate score spanning all domains [83, 91]. At this point, it remains unclear whether this finding can be attributed to an insufficient consideration of all domains or to uncertainties over how to weight and sum PL components to an overall score (for a call to ease administration and score PL calculation, see [93]). While the majority of interventions listed at least an outcome assessment related to the physical domain (physical competence), fewer articles included assessments related to the affective (motivation and confidence) and the cognitive (knowledge and understanding) domains (see Figs. 2, 3, and 4). This finding is in line with the analysis examining the design and content of PL interventions [29]. The combination of this review with the present analysis has the potential to show some nuances with respect to the cognitive and affective operationalization. While the affective domain appears to present a particular challenge for the translation of theory into interventional content, the insights on the cognitive domain rather point to difficulties in assessment. Against the background of these findings, researchers and practitioners might benefit from a more thorough consideration of suggestions from the general (e.g., [56]) and the PL-specific (e.g., [93–95]) literature on how to better cultivate these domains. As highlighted by a recent analysis of assessment instruments, the field could also profit from more holistic construction strategies by more consequently acknowledging the multifaceted nature of the concept [95]. In summary, a solution specific to PL interventions could lie in a more rigorous interlocking with PL theory at all stages of the whole intervention process, i.e., from the initial conceptualization and definition, via the formulation of intervention objectives and content to its concluding evaluation and interpretation [29].

Despite these negative statements in the previous section expressing the lack of theoretical-conceptual transfer, the meta-analysis shows that interventions adhering to PL exerted positive effects on all the main outcome categories of PL. Obviously, the interventional practices used so far are more successful in enhancing physical competence than knowledge and understanding or, in particular, motivation and confidence. In this context, a parallel can be identified between what is addressed (intervention content; see [29]) and what is finally measured (effectiveness). This, in turn, suggests that less holistic interventions (i.e., interventions targeting only a limited number of PL domains) may be less successful in achieving improvements in outcomes relevant for PA and health than interventions with a more holistic conceptualization (for a similar result with a specific perspective on motor skills, see [96]). At this point, however, caution is warranted as ongoing analyses in the PL context (e.g., meta-regression, moderator analysis) should corroborate these parallels first to prevent premature causal inferences being drawn. The strongest effect could be found for the physical competence domain, which harmonizes with content and concept analyses demonstrating that the PL literature places considerable emphasis on physical aspects [25, 97]. Physical literacy interventions also positively influenced individuals’ behavior by affecting their levels of PA. Taken together, the effect size registered in the present analysis was slightly higher than the aggregated effect size of an extensive review summarizing the effectiveness of theory-based PA interventions in general [57]. In this context, it cannot be excluded that the positive results may partially result from an expectancy effect simply reflecting participants’ interest in an innovative approach to PA and education. The sensitivity analysis implicated that differences in the effect size cannot be attributed to the circumstance that the present study included both randomized and non-randomized controlled trials. Comparisons between both study types could be performed for three of the five main categories, with none showing any meaningful differences. Rather, an explanation of this positive finding could be delivered by the fact that PL attempts to address multiple determinants of PA simultaneously. From a theoretical perspective, virtuous cycles are posited, describing reciprocal reinforcements between the different (e.g., affective, physical, behavioral) dimensions of PL [9, 35]. In any case, the results of the present review place the postulated theoretical links between PL and an increased engagement in physical activities [8, 9, 27, 35] on a stronger empirical basis.

Nevertheless, it was necessary to incorporate a multifaceted differentiation not only *between* the PL domains but also *within* the PL domains. While the motivation and confidence subcategories did not yield any meaningful differences with respect to the overall effect size, we recorded substantial differences within the knowledge and understanding domain. Most importantly, we identified considerable differences in the quantitative effect across the subcategories of physical competence. Whilst calculating small-to-moderate effects for cardiorespiratory fitness as well as agility and lower body strength, we observed large effect sizes for fundamental movement skills. On the one hand, this result may mirror that short-term improvements could be more easily achieved for technically dominated tasks (for similar effect sizes in a meta-analysis, see [98]) than for conditionally dominated tasks [99], especially when considering that most target groups consisted of children or adolescents. On the other hand, this finding may reflect that the term of fundamental movement skills enjoys remarkable popularity in the context of PL, in both research and practice [16, 100].

Despite these insights, we ascertained considerable heterogeneity and diversity across the different interventions. For instance, the included studies encompassed: (a) different age groups, from children in the early years [73] to adults in the older age [101]; (b) different target groups, from physically active individuals [102] to persons with clinical disorders [78]; (c) efforts from different cultures and continents; (d) different definitions and conceptualizations of PL (e.g., [14, 19, 21]); (e) different intervention deliverers, lengths, frequencies, and durations; (f) different research designs and study qualities (beyond the criterion of randomization); and (g) different assessment instruments, from self-reports [84] to objective measurements [103]. In general, this diversity can be evaluated from two different perspectives. From a methodological point of view, this circumstance can be considered as affecting the robustness of empirical findings. Against this background, research typically calls for a standardization of applications (e.g., intervention dose or assessment instruments) to promote the comparability of findings across studies. From a conceptual perspective, however, the diversity can be interpreted as an expression of the inclusive character, openness, and generalizability of the PL concept, benefiting practice in several populations and contexts. Given the heuristic value and the broad conceptualization of the PL approach and its domains [104, 105], the present findings should, therefore, be treated with a degree of caution. Nevertheless, the present review is, to our knowledge, the first study to apply a meta-analytical technique in the context of PL, with the results providing a complementary view on the concept and its interventional use.

In addition to the diversity (as mentioned above) inherent to the included primary studies, the present study has the following major limitations. First, our literature search focused on English articles only (language bias). This circumstance might have disproportionately favored intervention endeavors from Western and anglophone countries. Second, the search strategy concentrated exclusively on published articles, which may have prevented other relevant initiatives from entering the analyses (such as gray literature). Third, the analytical categories of the present study were driven by the IPLA [14] definition of PL. Among the different conceptualizations worldwide [23], this definition was chosen deliberately because the majority of PL literature recognizes the included domains. It cannot be excluded that the reliance on another definition [19, 21] may have yielded slightly different results (e.g., through the inclusion of a social or spiritual element). Fourth, the current findings barely provide empirically based recommendations on how to structure PL interventions. Meta-analytical moderator (subgroup) analyses or meta-regressions have the basic potential to extract those factors that significantly influence the effectiveness of interventions [69]. However, given the diversity across the studies, the extensive material reported in this article, and the fluctuating number of studies in the different (sub-)categories [69], it would have not been reasonable to basically perform these calculations. In this regard, the rapid developments of the field [25, 29], anticipating an increase in the number of studies in the next years, give hope that performing such important analyses will be possible in the near future. Last, the meta-analytical approach emphasized a quantitative perspective on PL interventions. The systematic review demonstrated that qualitative evaluations constitute an important part of PL interventions. Therefore, future studies should transcend the present approach by taking a specific qualitative view on PL interventions (e.g., by drawing on the methodology of qualitative meta synthesis [106]).

## Conclusions

The present findings highlight that an insufficient number of PL interventions are evaluated holistically. The field has considerable difficulties integrating operationalizations of knowledge and understanding as well as motivation and confidence. Therefore, future projects should strive for applying multidimensional assessments of PL to meet the holistic character of the concept. Simultaneously, the meta-analysis revealed that interventions with theoretical groundings in PL are effective in promoting outcomes of PL (despite some restraint given the significant publication bias). In this regard, PL appears to be highly attractive for practical endeavors in the context of PA. Importantly, because of the heuristic value and open character of the concept, researchers can apply PL in different populations, age groups, and settings [16]. Despite this flexibility, future interventions are recommended to cultivate a tight interlocking with PL theory at all stages of the intervention process. In the future, research should aim at identifying those program characteristics (moderators) that significantly influence the effectiveness of PL interventions.

## Supplementary Information

Below is the link to the electronic supplementary material.

Supplementary file1 (DOCX 546 KB)
