# Supplementary material for: The Effectiveness of Physical Literacy Interventions: A Systematic Review with Meta-Analysis
Source: Sports Med. 2022 Aug 22;52(12):2965–99. doi: 10.1007/s40279-022-01738-4 (PMC9691485; doi:10.1007/s40279-022-01738-4)
Supplement: Supplementary file 1 — Supplementary file1 (DOCX 546 KB) [file 40279_2022_1738_MOESM1_ESM.docx]

**Supplemental Material**

*Article:* The Effectiveness of Physical Literacy Interventions: A Systematic Review with Meta-Analysis

*Journal Name:* Sports Medicine

*Corresponding Author:* Dr. Johannes Carl (Friedrich-Alexander University Erlangen-Nürnberg, Department of Sport Science and Sport) – E-Mail Address: johannes.carl@fau.de

*Other Authors:* Jaime Barratt (The University of Queensland, School of Human Movement and Nutrition Sciences), Dr. Philipp Wanner (Ruprecht Karl University of Heidelberg, Institute of Sports and Sports Sciences), Dr. Clemens Töpfer (Friedrich Schiller University Jena, Institut of Sports Science), Prof. Dr. John Cairney (The University of Queensland, School of Human Movement and Nutrition Sciences), Prof. Dr. Klaus Pfeifer (Friedrich-Alexander University Erlangen-Nürnberg, Department of Sport Science and Sport).

**Table 1** PRISMA 2020 Checklist (Page et al., 2021)

| **Section and Topic** | **Item #** | **Checklist item** | **Location where item is reported** |
| --- | --- | --- | --- |
| **TITLE** | | |  |
| Title | 1 | Identify the report as a systematic review. | Page 1 |
| **ABSTRACT** | | |  |
| Abstract | 2 | See the PRISMA 2020 for Abstracts checklist. | Pages 4 and 5 |
| **INTRODUCTION** | | |  |
| Rationale | 3 | Describe the rationale for the review in the context of existing knowledge. | Pages 7-9 |
| Objectives | 4 | Provide an explicit statement of the objective(s) or question(s) the review addresses. | Page 9 |
| **METHODS** | | |  |
| Eligibility criteria | 5 | Specify the inclusion and exclusion criteria for the review and how studies were grouped for the syntheses. | Pages 11, 13 and 14 |
| Information sources | 6 | Specify all databases, registers, websites, organisations, reference lists and other sources searched or consulted to identify studies. Specify the date when each source was last searched or consulted. | Page 10 |
| Search strategy | 7 | Present the full search strategies for all databases, registers and websites, including any filters and limits used. | Page 10 and Appendix Table 2 |
| Selection process | 8 | Specify the methods used to decide whether a study met the inclusion criteria of the review, including how many reviewers screened each record and each report retrieved, whether they worked independently, and if applicable, details of automation tools used in the process. | Pages 10-12 |
| Data collection process | 9 | Specify the methods used to collect data from reports, including how many reviewers collected data from each report, whether they worked independently, any processes for obtaining or confirming data from study investigators, and if applicable, details of automation tools used in the process. | Pages 11 and 12 |
| Data items | 10a | List and define all outcomes for which data were sought. Specify whether all results that were compatible with each outcome domain in each study were sought (e.g. for all measures, time points, analyses), and if not, the methods used to decide which results to collect. | Pages 11 and 12 |
|  | 10b | List and define all other variables for which data were sought (e.g. participant and intervention characteristics, funding sources). Describe any assumptions made about any missing or unclear information. | Pages 11 and 12 |
| Study risk of bias assessment | 11 | Specify the methods used to assess risk of bias in the included studies, including details of the tool(s) used, how many reviewers assessed each study and whether they worked independently, and if applicable, details of automation tools used in the process. | Page 12 |
| Effect measures | 12 | Specify for each outcome the effect measure(s) (e.g. risk ratio, mean difference) used in the synthesis or presentation of results. | Pages 13 and 14 |
| Synthesis methods | 13a | Describe the processes used to decide which studies were eligible for each synthesis (e.g. tabulating the study intervention characteristics and comparing against the planned groups for each synthesis (item #5)). | Pages 11-12 |
|  | 13b | Describe any methods required to prepare the data for presentation or synthesis, such as handling of missing summary statistics, or data conversions. | Pages 13 and 14, Appendix Table 2 |
|  | 13c | Describe any methods used to tabulate or visually display results of individual studies and syntheses. | Page 14 |
|  | 13d | Describe any methods used to synthesize results and provide a rationale for the choice(s). If meta-analysis was performed, describe the model(s), method(s) to identify the presence and extent of statistical heterogeneity, and software package(s) used. | Pages 13 and 14 |
|  | 13e | Describe any methods used to explore possible causes of heterogeneity among study results (e.g. subgroup analysis, meta-regression). | Pages 13 and 14 |
|  | 13f | Describe any sensitivity analyses conducted to assess robustness of the synthesized results. | Page 14 |
| Reporting bias assessment | 14 | Describe any methods used to assess risk of bias due to missing results in a synthesis (arising from reporting biases). | Not performed |
| Certainty assessment | 15 | Describe any methods used to assess certainty (or confidence) in the body of evidence for an outcome. | Pages 13 and 14 |
| **RESULTS** | | |  |
| Study selection | 16a | Describe the results of the search and selection process, from the number of records identified in the search to the number of studies included in the review, ideally using a flow diagram. | Page 15, Figure 1 |
|  | 16b | Cite studies that might appear to meet the inclusion criteria, but which were excluded, and explain why they were excluded. | See three-step reporting approach (Pages 14-21) |
| Study characteristics | 17 | Cite each included study and present its characteristics. | Tables 1 and 2 |
| Risk of bias in studies | 18 | Present assessments of risk of bias for each included study. | Page 16, Table 3 |
| Results of individual studies | 19 | For all outcomes, present, for each study: (a) summary statistics for each group (where appropriate) and (b) an effect estimate and its precision (e.g. confidence/credible interval), ideally using structured tables or plots. | Figures 2, 3, and 4 |
| Results of syntheses | 20a | For each synthesis, briefly summarise the characteristics and risk of bias among contributing studies. | Pages 16-21 |
|  | 20b | Present results of all statistical syntheses conducted. If meta-analysis was done, present for each the summary estimate and its precision (e.g. confidence/credible interval) and measures of statistical heterogeneity. If comparing groups, describe the direction of the effect. | Pages 16-21 Figures 2, 3, and 4 |
|  | 20c | Present results of all investigations of possible causes of heterogeneity among study results. | Pages 16-21 Figure 2, 3, and 4 |
|  | 20d | Present results of all sensitivity analyses conducted to assess the robustness of the synthesized results. | Pages 16 and 17 |
| Reporting biases | 21 | Present assessments of risk of bias due to missing results (arising from reporting biases) for each synthesis assessed. | Not performed |
| Certainty of evidence | 22 | Present assessments of certainty (or confidence) in the body of evidence for each outcome assessed. | Pages 16-21 Figures 2, 3, and 4 |
| **DISCUSSION** | | |  |
| Discussion | 23a | Provide a general interpretation of the results in the context of other evidence. | Pages 21-23 |
|  | 23b | Discuss any limitations of the evidence included in the review. | Pages 24 and 25 |
|  | 23c | Discuss any limitations of the review processes used. | Pages 24 and 25 |
|  | 23d | Discuss implications of the results for practice, policy, and future research. | Pages 21-26 |
| **OTHER INFORMATION** | | |  |
| Registration and protocol | 24a | Provide registration information for the review, including register name and registration number, or state that the review was not registered. | Pages 9 and 10 |
|  | 24b | Indicate where the review protocol can be accessed, or state that a protocol was not prepared. | Pages 9 and 10 |
|  | 24c | Describe and explain any amendments to information provided at registration or in the protocol. | Page 10 |
| Support | 25 | Describe sources of financial or non-financial support for the review, and the role of the funders or sponsors in the review. | Page 26 |
| Competing interests | 26 | Declare any competing interests of review authors. | Page 27 |
| Availability of data, code and other materials | 27 | Report which of the following are publicly available and where they can be found: template data collection forms; data extracted from included studies; data used for all analyses; analytic code; any other materials used in the review. | All material that is not part of the main article or the appendix can be retrieved upon request |

*From:*  Page MJ, McKenzie JE, Bossuyt PM, Boutron I, Hoffmann TC, Mulrow CD, et al. The PRISMA 2020 statement: an updated guideline for reporting systematic reviews. BMJ 2021;372:n71. doi: 10.1136/bmj.n71

For more information, visit: <http://www.prisma-statement.org/>

**Table 2** The successive development of the final search strategy.

| ***Exploratory Searches*** | |  |
| --- | --- | --- |
| 01 | “physical literacy” | 401^A^ |
| 02 | (interven* OR program* OR evaluat* OR effect*) |  |
| 03 | 01 AND 02 | 181^A^ |
| 04 | (interven* OR program* OR evaluat* OR effect* OR efficacy OR protocol OR promot* OR workshop OR educat*) |  |
| 05 | 01 AND 04 | 206^A^ |
| 06 | (interven* OR program* OR evaluat* OR effect* OR efficacy OR protocol OR promot* OR workshop OR educat* OR training) |  |
| 07 | 01 AND 06 | 381^A^ |
| 08 | (interven* OR program* OR evaluat* OR effect* OR efficacy OR protocol OR promot* OR workshop OR educat* OR training OR treat* OR trial OR experiment*) |  |
| 09 | 01 AND 08 | 384^A^ |
| 10 | „physically literate“ |  |
| 11 | (01 OR 10) AND 08 | 407^A^ |
| 12 | (interven* OR program* OR evaluat* OR effect* OR efficacy OR protocol OR promot* OR workshop OR educat* OR training OR treat* OR trial OR experiment* OR exercis*) |  |
| 13 | (01 OR 10) AND 12 | 411^A^ |
| ***Summary: Final Search Term Combination with Truncations:***  *("physical literacy" OR "physically literate") AND (interven* OR program* OR evaluat* OR promot* OR workshop OR treat* OR trial OR educat*OR evaluat* OR protocol OR training OR effect* OR efficacy OR experiment* OR exercis*)* | | |
| ***Summary: Final Search Term Combination without Truncations:***  *("physical literacy" OR "physically literate") AND (intervention OR interventions OR program OR programme OR programs OR programmes OR evaluate OR evaluation OR promotion OR promote OR promoting OR workshop OR treating OR treat OR treatment OR trial OR educate OR education OR evaluative OR protocol OR training OR effect OR effectiveness OR efficacy OR effective OR experiment OR experimental OR exercise OR exercising)* | | |

*^A^Exemplarily showing the initial number of hits gathered in the meta-database EBSCO, including the sub-databases Psychology and Behavioral Sciences Collection, SPORTDiscus, APAPsycINFO, Teacher Reference Center, APA PsycARTICLES.*

**Table 3** Explanations for the Introduction of the Meta-analytical Subcategories.

| **Main Category** | **Explanation** |
| --- | --- |
| Physical Competence | The domain “physical competence” was further split into *three subgroups*. In any case, it should be considered whether tasks, and therefore individual capabilities, are more skill-oriented or more energetically determined. **Fundamental movement skills** are discussed and used very frequently in the physical literacy literature [1–4]. Accordingly, covering the more technically/skill-oriented side of physical competence, these were awarded an own subcategory. In the area of energetically determined capabilities, the variables identified across the studies induced us to bundle different endurance-oriented indicators (thus termed **“cardiorespiratory fitness”**) and indicators representing speed-/strength-oriented capabilities of the lower extremity (thus termed **“agility and lower body strength”**). In this regard, the categorization in the area of energetically determined capabilities accounted for the need to differentiate between movements with high intensities, short durations, and a stronger involvement of type IIa/x muscle fibers as well as movements with low intensities, long durations, and a predominant involvement of type I muscle fibers [5]. The number of other indicators were not large enough [6] to form further subcategories (e.g., related to upper-body strength or flexibility). A detailed overview of the meta-analytical results of this main category and its subgroups is given in Table 2. |
| Knowledge and Understanding | The domain “knowledge and understanding” was further split into *two subgroups*. This subgroup is also often called the “cognitive” domain [3, 7, 8]. On the one hand, cognitive demands can refer to the **object** of physical activity, exercise, fitness, and sport (e.g., general health-related effects or the proper execution of exercises) independent from the individual experience (e.g., [9, 10]). These cognitions can be briefly described as covering the “knowledge” aspect of the domain. On the other hand, cognitions can refer to one’s **subjectively constructed relation** to physical activity, exercise, fitness, and sport, with the own perspective (e.g., meaning making, personal attitude) standing in the center of interest (e.g., [11, 12]). These cognitions can be briefly described as covering the “understanding” aspect of the domain. The side to which a cognitive indicator can be assigned, in turn, often suggests the modality how data is typically acquired (e.g., standardized test versus self-evaluation). Accordingly and in line with the material/indicators found, we split up between **“objective knowledge”** as well as **“subjective understanding and attitude”**. A detailed overview of the meta-analytical results of this main category and its subgroups is given in Table 4. |
| Motivation and Confidence | The domain “motivation and confidence” was further split into *three subgroups*. In accordance with the designation of this domain, we cultivated a differentiation into the subgroups motivation and confidence. **Motivation** characterizes “the impetus that gives purpose or direction to [physical activity] behavior” [13] and served as the first subgroup. Moreover, a second subgroup was formed with **confidence** that can be defined as the “belief that one is capable of successfully meeting the demands of a[n activity-related] task” [13]. In this context, we also included the related concept of **self-efficacy** as a frequent and related [14] operationalization of this domain. In addition to these more time-invariant (stable) concepts on the personal level, the analysis revealed that the domain is several times operationalized via the state-like variables **affect** or **enjoyment**. This is line with the fact that this domain is also often called the “affective domain” [3, 7]. A detailed overview of the meta-analytical results of this main category and its subgroups is given in Table 3. |

Note: As mentioned in the main file, subgroups were formed after familiarization with the extracted PL indicators (which implicates a material-driven or inductive approach, respectively).

**Literature of the Table:**

1. Tompsett C, Burkett B, McKean MR. Development of Physical Literacy and Movement Competency: A Literature Review. Journal of Fitness Research. 2014;3:53–74.

2. Hulteen RM, Morgan PJ, Barnett LM, Stodden DF, Lubans DR. Development of foundational movement skills: A conceptual model for physical activity across the lifespan. Sports Med. 2018;48:1533–40. doi:10.1007/s40279-018-0892-6.

3. Edwards LC, Bryant AS, Keegan RJ, Morgan K, Jones AM. Definitions, Foundations and Associations of Physical Literacy: A Systematic Review. Sports Med. 2017;47:113–26. doi:10.1007/s40279-016-0560-7.

4. Young L, O’Connor J, Alfrey L. Physical literacy: a concept analysis. Sport, Education and Society. 2020;25:946–59. doi:10.1080/13573322.2019.1677586.

5. Haff GG, Triplett NT. Essentials of strength training and conditioning 4th edition: Human Kinetics; 2015.

6. Higgins J, Green S. Cochrane Handbook for Systematic Reviews of Interventions (Cochrane Book Series). Chichester, UK: Joh Wiley & Sons; 2008.

7. Keegan RJ, Barnett LM, Dudley DA, Telford RD, Lubans D, Bryant AS, et al. Defining Physical Literacy for Application in Australia: A Modified Delphi Method. Journal of Teaching in Physical Education. 2019;38:105–18. doi:10.1123/jtpe.2018-0264.

8. Shearer C, Goss HR, Boddy LM, Knowles ZR, Durden-Myers EJ, Foweather L. Assessments Related to the Physical, Affective and Cognitive Domains of Physical Literacy Amongst Children Aged 7-11.9 Years: A Systematic Review. Sports Medicine - Open. 2021;7:37. doi:10.1186/s40798-021-00324-8.

9. Demetriou Y, Sudeck G, Thiel A, Höner O. The effects of school-based physical activity interventions on students' health-related fitness knowledge: A systematic review. Educational Research Review. 2015;16:19–40. doi:10.1016/j.edurev.2015.07.002.

10. Chen S, Liu Y, Schaben J. To move more and sit less: Does physical activity/fitness knowledge matter in youth? Journal of Teaching in Physical Education. 2017;36:142–51.

11. French DP, Sutton S, Hennings SJ, Mitchell J, Wareham NJ, Griffin S, et al. The importance of affective beliefs and attitudes in the theory of planned behavior: predicting intention to increase physical activity 1. Journal of Applied Social Psychology. 2005;35:1824–48.

12. Silverman S, Subramaniam PR. Student attitude toward physical education and physical activity: A review of measurement issues and outcomes. Journal of Teaching in Physical Education. 1999;19:97–125.

13. American Psychological Association. APA Dictionary of Psychology. 2022. https://dictionary.apa.org/.

14. Holler P, Jaunig J, Moser O, Tuttner S, Simi H, Wallner D, et al. Primary Care and Physical Literacy: A Non-Randomized Controlled Pilot Study to Combat the High Prevalence of Physically Inactive Adults in Austria. IJERPH. 2021;18:8593. doi:10.3390/ijerph18168593.

**Table 4** Details regarding the processing or adjustment of information for the meta-analysis.

| **Study (Sample)** | **Reason for further proceeding** | **Calculated values*** | **Data used in the Review Manager (see also forest plots)** |
| --- | --- | --- | --- |
| Bremer et al., 2020 [80] | Contact with author due to not reporting post-test values, significant baseline differences for movement competence | Baseline-adjusted PLAYfun average score SMD -0.21 | Adjustment of the control group mean for movement competence (original as reported in the contact with the author: 52.5; adjusted for baseline: 45.0) |
| Choi et al., 2021 [90] | Two indicators for motivation (intrinsic motivation and identified motivation) | Intrinsic SMD 0.18 Identified SMD 0.12  🡪 Mean SMD 0.15 | Use of the intrinsic motivation value, adjustment of the control group mean (original: 20.22; adjusted: 20.36) |
| Coutinho et al., 2018 [102] – U15 | Three different indicators for “agility and lower body strength” | Sprint 30m SMD -0.38 Change of Direction Task SMD 0.69 Jump SMD 0.06 🡪 Mean SMD 0.13 | Use of the countermovement jump values, adjustment of the control group mean (original: 35.01; adjusted: 31.93) |
| Coutinho et al., 2018 [102] – U17 | Three different indicators for “agility and lower body strength”, wrong values reported in manuscript (authors were contacted) | Sprint 30m SMD 0.62 Change of Direction Task SMD 0.66 Jump SMD 0.68 🡪 Mean SMD 0.65 | Use of the countermovement jump values, adjustment of the control group mean (original: 37.63; adjusted: 30.62) |
| Guerrero & Chandler, 2018 [79] | Two indicators for motivation | Autonomous motivation SMD 3.01 Controlled motivation (despite inverting) SMD -2.87 🡪 Mean SMD 0.12 | Use of the autonomous motivation values, adjustment of the control group mean (original: 3.48; adjusted: 4.61) |
| Kriellaars et al., 2019 [91] | Effect sizes for physical activity had to be estimated from mean values, significance level and sample sizes | Mean intervention group 26.0 (*n* = 78) Mean control group 20.0 (*n* = 70) Significance treatment effect *p* = 0.05 (p. 166) | Physical activity pursuits SMD 0.34 |
| Kwan et al., 2019, 2020 [34, 92] | Marginally significant baseline differences for cardiovascular fitness (*p* = .07) and lower body strength (*p* = .01) | Adjusted effects for cardiovascular fitness SMD 0.38 and lower body strength SMD -0.07 | Adjustment of the control group mean for fitness (original: 40.22; adjusted: 42.60) and strength (original: 172.6; adjusted: 195.2) |
| Mateus et al., 2015 [103] | No information about potential baseline differences | Baseline-adjusted SMD for agility 0.31 | Inverting and adjustment of the control group mean for fitness (original: 15.2; adjusted: 15.5) |
| Pullen et al., 2020 [123] – Females | Only median and interquartile values indicated, transformation into standard deviation necessary (despite initial contact, author did not provide further information) | Motor skills SMD 1.09 Autonomous Motivation SMD 0.21 Self-Efficacy SMD 0.37 | Intervention group mean 26.0 SD 4.46  Control group mean 21.0 SD 4.46 |
| Pullen et al., 2020 [123] – Males |  | Motor skills SMD 1.26 Autonomous Motivation SMD 0.32 Self-Efficacy SMD 0.45 | Intervention group mean 26.0 SD 3.53  Control group mean 41 SD 4.08 |
| Santos et al., 2017 [124] | Three indicators for “agility and lower body strength” | Jump SMD 0.20 Speed SMD 0.23  Agility SMD 1.79 🡪 Mean SMD 0.74 | Use of the jump value, adjustment of the control group mean (original: 19.80; adjusted: 22.23) |
| Telford et al., 2020 [49] | Two indicators for fundamental movement skills | Object control SMD 0.31  Locomotor control SMD 0.12 🡪 Mean SMD 0.21 | Use of an averaged mean: Intervention group mean 36.66 SD 5.14, control group mean 36.06 (adjusted to 35.55 in line with SMD) SD 5.18 |
| Wright et al., 2020 [132] | Five indicators of fundamental movement skills | Run SMD 0.21 Hop SMD 0.20 Overhand SMD 0.50 Kick ball SMD 0.15 Balance SMD 0.13 🡪 Mean SMD 0.24 | Use of an averaged mean: Intervention group mean 54.20 SD 8.90, control group mean 49.98 (adjusted to 52.25 in line with SMD) SD 7.22 |

*Note:* *Effect Sizes were computed via Psychometrica – Operation 3 for Pre-Post-Control Designs [27]
Abbreviations: SMD = standardized mean difference; SD = standard deviation.

**Table 5** Results of the sensitivity analysis.

| **Main Category** | **Randomized controlled trials** | | **Non-randomized controlled trials** | | **Test for subgroup differences** |
| --- | --- | --- | --- | --- | --- |
|  | *k* | SMD [95%CI] | *k* | SMD [95%CI] |  |
| Physical Competence | 11 | 1.02 [0.49, 1.55] | 10 | 0.66 [0.22, 1.11] | *χ*²(1) = 1.00, *p* = 0.32; *I*² = 0.2% |
| Motivation and Confidence | 7 | 0.30 [0.11, 0.49] | 7 | 0.32 [0.16, 0.48] | *χ*²(1) = 0.03, *p* = 0.87; *I*² = 0% |
| Knowledge and Understanding | 2 | 1.10 [-0.93, 3.13] | 5 | 0.50 [0.32, 0.68] | Test not performed due to an insufficient number of samples across both categories (even for exploratory analysis) |
| Physical Activity Behavior | 5 | 0.39 [0.13, 0.65] | 6 | 0.41 [0.21, 0.61] | *χ*²(1) = 0.01, *p* = 0.92; *I*² = 0% |
| Total PL Score | 2 | 0.72 [-0.67, 2.11] | 3 | 0.63 [0.32, 0.94] | Test not performed due to an insufficient number of samples across both categories (even for exploratory analysis) |

Note: *k* = number of different samples included (not outcomes); SMD = standardized mean difference.

**Fig. 1** Funnel plots.


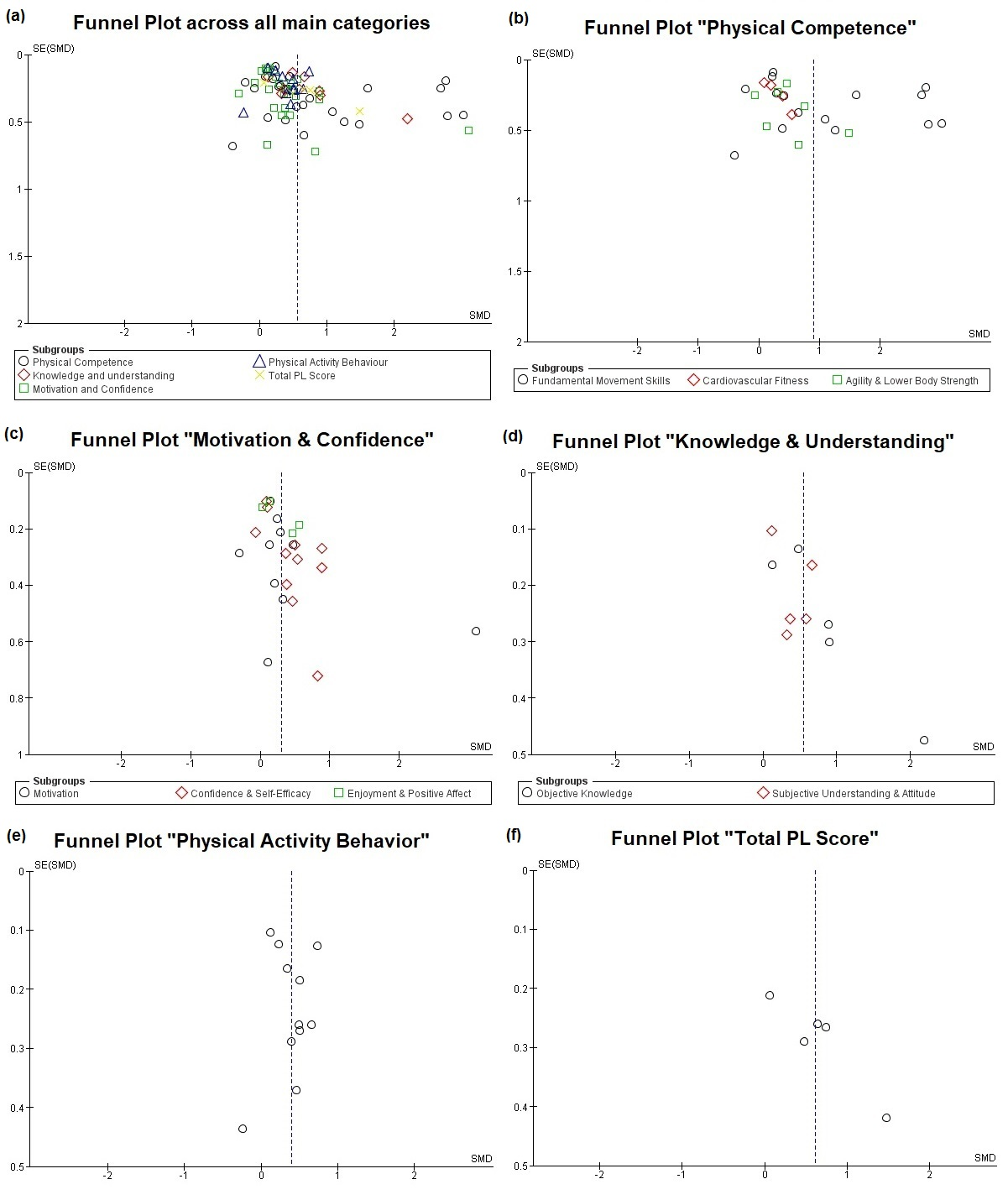

Note: Visual inspection of the asymmetry of the different funnel plots according to different analytical categories: (a) all main categories; (b) main category “physical competence”; (c) main category “motivation and confidence”; (d) main category “knowledge and understanding”; (e) main category “physical activity behavior”; (f) main category “total PL score”.
Abbreviations: PL = Physical Literacy, SE = Standard Error, SMD = Standardized Mean Difference.
